# Beyond Spin Crossover: Optical and Electronic Horizons of 2,6-Bis(pyrazol-1-yl)pyridine Ligands and Complexes

**DOI:** 10.3390/molecules30061314

**Published:** 2025-03-14

**Authors:** Yuliia Oleksii, Abdelkrim El-Ghayoury

**Affiliations:** Univ Angers, CNRS, MOLTECH—Anjou, SFR MATRIX, F-49000 Angers, France; yuliia.oleksii@univ-angers.fr

**Keywords:** 2,6-bis(pyrazol-1-yl)pyridine (**bpp**), spin crossover (SCO), luminescence, supramolecular chemistry, photonic devices

## Abstract

The 2,6-bis(pyrazol-1-yl)pyridine (**bpp**) ligand family is widely recognized for its versatile coordination abilities and broad functionalization potential. This review examines **bpp** and its modifications at the pyridine ring’s 4-position, focusing on their influence on magnetic, optical, and electronic properties. Key applications discussed include spin crossover (SCO), single-ion and single-molecule magnetism (SIM and SMM), luminescence, redox flow batteries (RFBs), and photonic devices. We provide a comprehensive overview of ligand modifications involving carboxylates, extended aromatic systems, radicals, and redox-active units such as tetrathiafulvalene (TTF), alongside supramolecular architectures. The review highlights fundamental design principles, particularly the role of substituents in tuning the SCO behavior, photophysical properties, and self-assembly into functional nanostructures. Notable advancements include SCO-driven conductivity modulation, reversible luminescent switching, and amphiphilic **bpp**-based vesicles for multicolor emission. By analyzing the interplay between ligand structure and magnetic, optical, and electronic functions, we provide insights into the potential of **bpp** derivatives for advanced materials design. This review presents recent experimental and theoretical developments, offering a foundation for future exploration of **bpp**-based compounds in multifunctional devices.

## 1. Introduction

**Figure 1:**
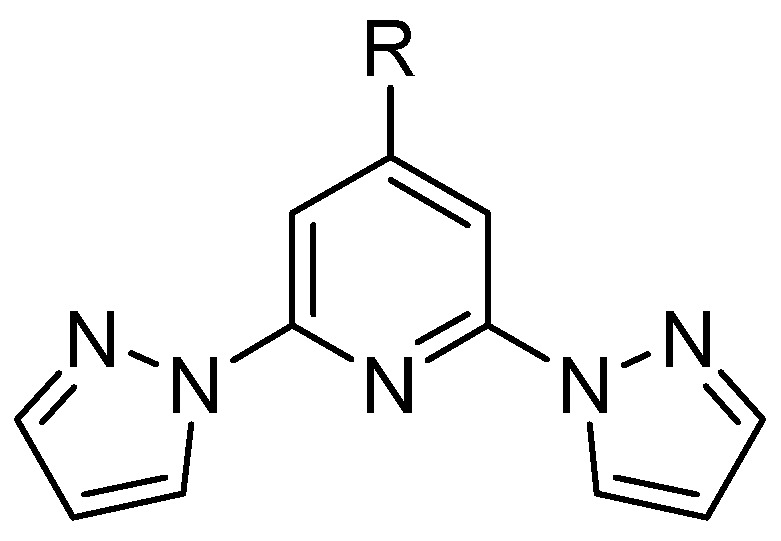
Ligand type discussed in this work.

2,6-Bis(pyrazol-1-yl)pyridine (**bpp**) and its derivatives continue to capture broad attention in coordination chemistry owing to their robust tridentate N-donor framework, straightforward syntheses, and extensive functionalization possibilities at the pyridine ring’s 4-position (Figure 1). Indeed, from simple alkyl and halogen substituents to extended aromatic systems, vinyl bridges, alkynyl linkers, or carboxylic/ester moieties, the 4-position has proven invaluable for building multifunctional architectures [1,2].

Historically, **bpp** complexes are perhaps best known for their spin crossover (SCO) properties with iron(II), and many seminal discoveries in that domain have already been reviewed extensively [3,4,5]. While SCO remains a pivotal facet of **bpp** research, this review focuses on other important functionalities and applications—ranging from luminescence and single-ion/single-molecule magnetism (SIM, SMM) to redox flow batteries (RFBs), surface anchoring strategies, and photonic devices. The broad chemical versatility of **bpp** ligands is further exemplified by their applications in catalysis, including olefin [6] and butadiene [7,8] polymerization, alkene oxidation [9], hydroboration [10], cross-coupling [11,12] reactions, and cycloaddition of CO_2_ to epoxides [13]. While we do not explore these studies in detail, they highlight the utility of **bpp** scaffolds in assembling reactive metal centers.

Notably, not all **bpp** systems rely on coordination to metals. In “back-to-back” **bpp** architectures, for example, purely organic **bpp**-based molecules can assemble into waveguides, nanotubes, and other photonic devices that exhibit multicolor luminescence or facilitate light propagation without ever forming metal complexes [14]. These materials derive their functionality from the **bpp** core’s π–π interactions, hydrogen bonding, and structural rigidity, yielding fascinating self-assemblies that operate as optical waveguides, fluorescent “microtubes”, and even flexible photonic circuits.

A key aim of this review is to underscore how relatively simple modifications at the 4-position of the pyridine ring can enable covalent attachment of diverse functional units—via single C–C bonds, vinyl linkages, acetylenic bridges—and, in turn, unlock new avenues for tuning magnetic, optical, or electronic behavior. To provide clarity, we structured the content into sections according to the specific substitution or functional group introduced at the 4-position (e.g., carboxylates, extended aromatics, tetrathiafulvalene (TTF) units), and, whenever feasible, we present the advances in a roughly chronological manner to reveal how each family of **bpp** derivatives has evolved over time. By focusing on the emergent properties and applications associated with both metal-coordinated and purely organic **bpp** derivatives —and how even modest structural modifications can reshape their behavior—we aim to illustrate the remarkable adaptability of the **bpp** motif in advanced materials design, far beyond its frequently cited role in SCO.

## 2. Foundations and Versatility of bpp Complexes

**Figure 2:**
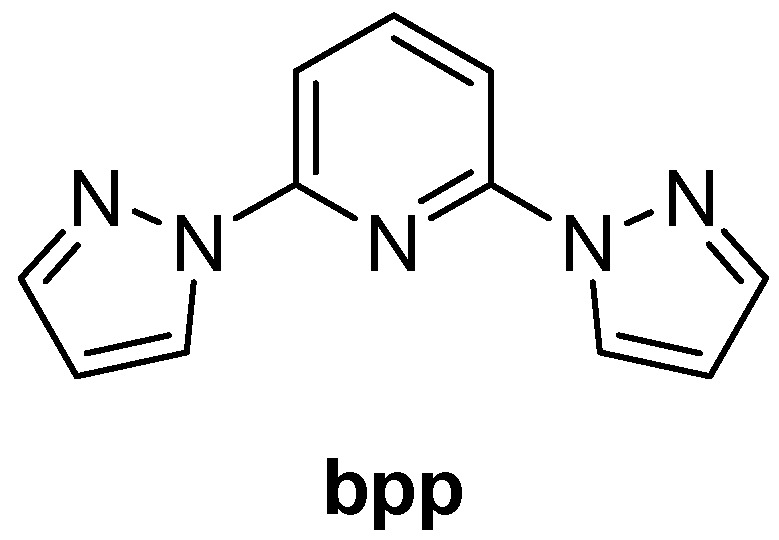
Structure of the **bpp** ligand discussed in Section 2.

The **bpp** ligand has emerged as a versatile building block in coordination chemistry, offering a tridentate N-donor framework suitable for a broad spectrum of metal complexes (Figure 2). Researchers have explored its spectroscopic and electronic features in considerable detail, and its complexes now encompass diverse fields such as SCO, luminescence, RFBs, SIM, and photovoltaic devices. In what follows, the core structural and electronic traits of the **bpp** ligand are briefly outlined, followed by a chronological overview of key studies on d- and f-metal complexes, with special emphasis on the synergy between SCO and other physical properties whenever it arises.

### 2.1. Structural/Spectroscopic Foundations of **bpp**

Early detailed insights into the electronic and spectroscopic properties of the **bpp** ligand came from the work of Adeniyi et al. in 2014 [15]. In that study, they synthesized and characterized multiple pyrazole derivatives, with particular attention given to the **bpp** system. The synthesis of **bpp** was achieved using a simple method combining the existing approaches [16,17] without requiring inert conditions or long reaction times. The reaction of pyrazole with dichloropyridine in a 6:1 molar ratio, using potassium carbonate, potassium hydroxide, and benzyltriethylammonium chloride as a phase transfer catalyst, produced **bpp**, which was purified by means of acid precipitation and filtration. Their combination of experimental (IR, Raman, NMR) and theoretical (time-dependent density functional theory (TDDFT)) approaches revealed strong correlations between the measured and calculated vibrational frequencies (R^2^ of up to 0.997) and a similarly tight agreement between the experimental and computed NMR shifts. The high level of agreement validated the structural assignments of **bpp** and the related pyrazole-based ligands. They further found that among the tridentate ligands they studied, **bpp** possessed relatively high conductivity and hyperpolarizability parameters, highlighting its promise in nonlinear optical applications and in materials where good charge transport is required. The article’s novelty lay in coupling thorough spectroscopic characterization with predictive quantum-chemical calculations, thereby establishing a strong foundation for rational ligand design.

### 2.2. Iron-Based **bpp** Complexes: Coupling Spin-State Behavior with Multifunctional Properties

Iron complexes incorporating **bpp** came under scrutiny, particularly for their spin state behavior. In 2000, Ayers et al. [18] provided an early examination of [Fe(**bpp**)_2_](PF_6_)_2_. Magnetic measurements indicated an effective magnetic moment μ_eff_ of around 4.6 B.M. at room temperature, suggesting a largely high-spin (HS) Fe(II) configuration (d^6^). The Fe(III/II) redox process showed irreversible features that the authors attributed to large geometric reorganizations induced by oxidation and partial spin state changes. Although the complex did not display a classic fully switchable spin transition, they identified a borderline regime between HS and low-spin (LS) states. The novelty at the time arose from systematically comparing ligands that replace pyridine donors with pyrazoles in robust tridentate frameworks. The weaker ligand field imposed by pyrazoles shifted iron complexes toward higher-spin states and more anodic redox potentials, revealing how subtle alterations in the donor set can tune both spin state and redox behavior in a predictable way.

**Figure 3:**
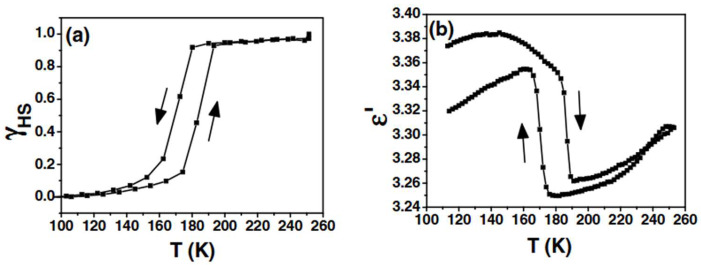
(**a**) Thermal variation of the proportion of high-spin molecules (γ_HS_) in [Fe(**bpp**)_2_](BF_4_)_2_ obtained through magnetic susceptibility measurements. (**b**) Thermal hysteresis of the dielectric constant associated with the spin transition of [Fe(**bpp**)_2_](BF_4_)_2_. The arrows indicate the heating and cooling cycles during the measurements. Reprinted with permission from [19], Copyright (2006), Wiley.

A more unambiguous example of iron-based SCO was investigated by Guillon et al. in 2006 [19]. Focusing on [Fe(**bpp**)_2_](BF_4_)_2_, they tracked a thermal SCO near 180 K with about 16 K of hysteresis (Figure 3a). By measuring the real part of the dielectric constant (ε′), they discovered that the dielectric response closely followed the SCO transition but with an unusual sign, showing a higher ε′ in the LS phase (Figure 3b), contrary to many Fe(II) SCO systems for which the HS phase is more polarizable [20]. Single-molecule density functional theory (DFT) calculations indicated a higher polarizability for the HS species, which conflicted with the experimental observation unless counteranions and intermolecular packing were explicitly considered. This underscored how crystal packing and anion interactions must be included in realistic models of the bulk dielectric response. The study’s novelty lay in linking macroscopic dielectric behavior to the spin state, suggesting possible capacitor or memory applications based on SCO.

**Figure 4:**
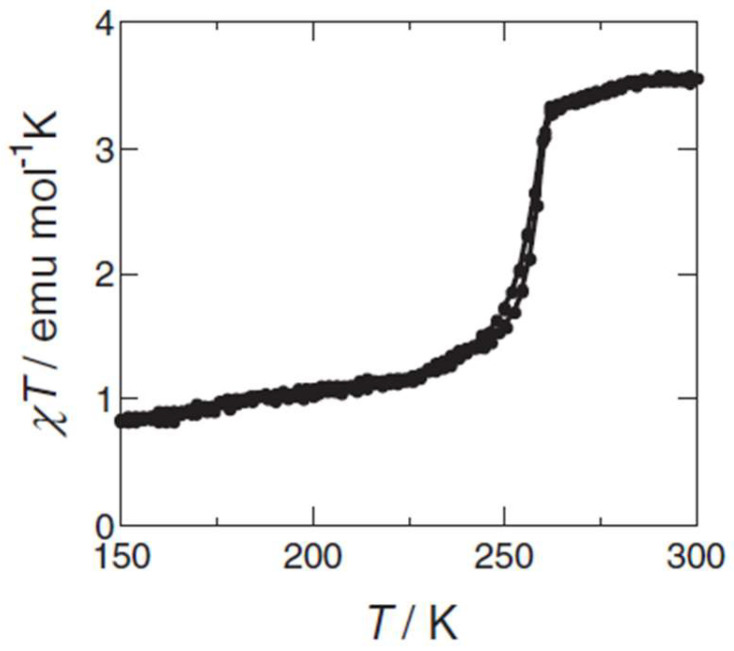
Plots of χT vs. T for [Fe(**bpp**)_2_](BF_4_)_2_ during cooling and heating processes. Reprinted with permission from [21], Copyright (2007), The Chemical Society of Japan.

Building on such findings, Matsuda and Tajima led multiple studies from 2007 to 2013 that explored the synergy between SCO and other properties in iron complexes based on the **bpp** ligand. In 2007 [21], they demonstrated that [Fe(**bpp**)_2_](BF_4_)_2_ could be spin-coated onto substrates to form smooth thin films (~30 nm). The thermally driven spin transition remained abrupt near 260 K (Figure 4), and they discovered that the film’s electrical resistance changed by ~25% between the HS and LS states. In 2008 [22,23], they blended the same [Fe(**bpp**)_2_](BF_4_)_2_ complex with chlorophyll a in an organic electroluminescent device. At temperatures above ~260 K, the iron complex was HS, permitting normal electroluminescence (EL). Below the spin transition, however, the LS state almost completely quenched the electroluminescence emission. This on/off emission switching was reversible over multiple cycles, indicating a direct synergy between the SCO transition and carrier injection mechanisms. Subsequent work in 2013 [24] refined the mechanistic understanding by showing that energy level shifts in the iron center modulated electron injection and transport, especially in the LS form. The series of studies proved that spin transitions in **bpp** iron complexes can be harnessed to gate electronic and luminescent properties, highlighting a unique route toward multifunctional optoelectronic devices.

Electrical gating of single **bpp**-containing iron complexes was further explored by Meded et al. in 2011 [25]. They reported a single-molecule junction experiment where the Fe(II)–**bpp** system switched its spin state as a function of electrostatic gating. This triggered changes in the Kondo resonance, reflecting how conduction phenomena and SCO are deeply coupled at the molecular level. The novelty was in demonstrating direct electrical control over the spin state, paving the way for spintronic applications.

**Figure 5:**
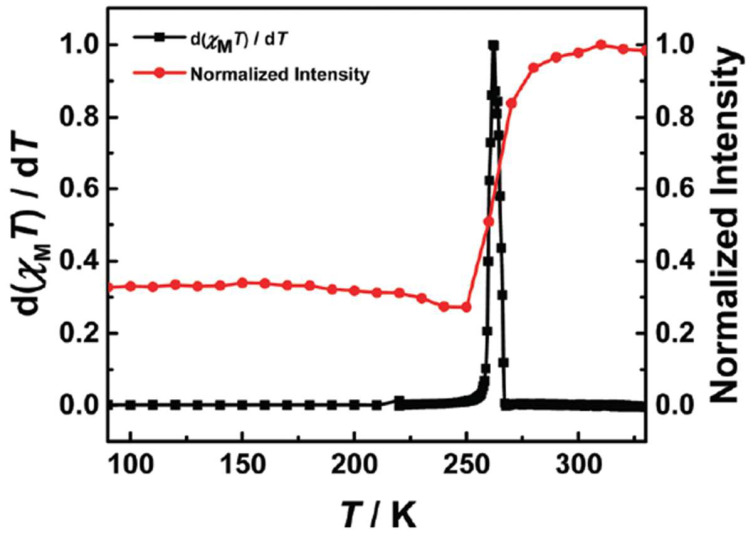
Normalized luminescence intensity at 493 nm and differential magnetic susceptibility (d(χ_M_T)/dT) of [Fe(**bpp**)_2_][BF_4_]_2_ in the heating mode from 90 to 330 K. Reprinted with permission from [26], Copyright (2017), Royal Society of Chemistry.

In 2017, Jiao et al. documented a remarkable synergy between SCO and luminescence in [Fe(**bpp**)_2_](BF_4_)_2_ [26]. In contrast to many Fe(II) systems where HS states often quench emission, here, the HS phase around 260 K correlated with a pronounced luminescence “turn-on” (Figure 5). The authors attributed this uncommon behavior to a favorable metal-to-ligand charge transfer (MLCT) pathway that became more accessible in the HS configuration, thereby producing an SCO-driven light emission switch. The novelty was the direct observation of luminescence switching that depends on the spin transition within the very same iron compound, rather than relies on an external fluorophore.

**Figure 6:**
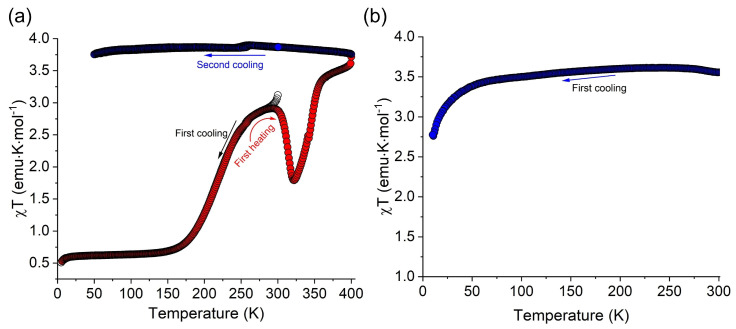
(**a**) The temperature dependence of the χT product of complex [Fe(**bpp**)_2_](TCNQ)_3.5_·3.5MeCN measured on a freshly prepared polycrystalline sample. (**b**) The temperature dependence of the χT product of complex [Fe(**bpp**)_2_](TCNQ)_3.5_·3.5MeCN measured on a sample dried under vacuum for 12 h prior to the measurements. Reprinted with permission from [27], Copyright (2021), Wiley.

A more recent angle on iron-based **bpp** complexes comes from the solvent-dependent semiconducting behavior studied by Üngör et al. in 2021 [27]. Their cocrystals combined fractional 7,7,8,8-tetracyanoquinodimethane (TCNQ) radical anions with [Fe(**bpp**)_2_]^2+^, yielding materials that showed both electrical conductivity (~10^−^^4^–10^−^^3^ S/cm) and gradual spin transitions, but only when sufficiently solvated. Upon solvent loss, the crystal lattice collapsed into a fully HS iron with no SCO left (Figure 6), yet the sample remained semiconducting. Although the synergy between SCO and conduction was partially obscured by solvent effects, the work nonetheless advanced efforts to merge spin state switching and charge transport in integrated hybrid materials.

### 2.3. Cobalt-Based **bpp** Complexes: Redox Activity, Magnetism, and Energy Storage

**Figure 7:**
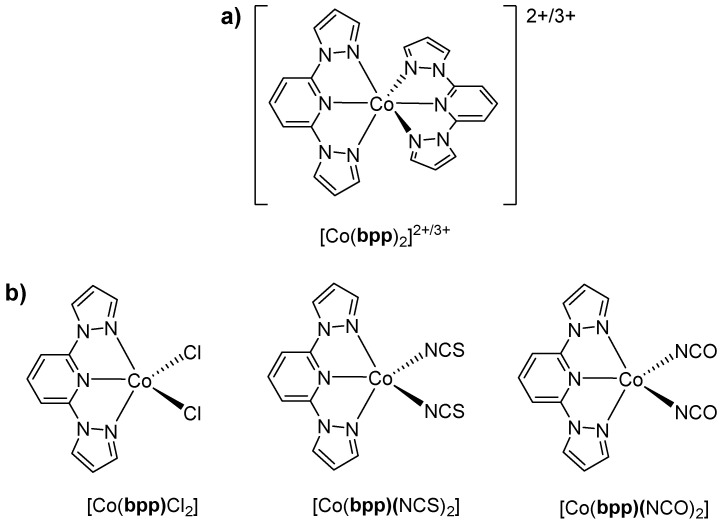
(**a**) Structure of the [Co(**bpp**)_2_]^2+/3+^ complex described in [18,28,29,30,31]. (**b**) Structures of the Co(II) complexes described in [32,33].

Beyond iron, several cobalt–**bpp** systems have also attracted attention. In an already mentioned publication [18], it was found that [Co(**bpp**)_2_](PF_6_)_2_·H_2_O (Figure 7a) had a purely HS d^7^ configuration (μ_eff_ = 4.6 B.M.), stabilized by the ligand’s moderate field strength. They noted a quasi-reversible Co(III/II) redox couple that shifted anodically relative to the complexes bearing more pyridines, again demonstrating how partial replacement with pyrazole donors lowers the ligand field. Although spin crossover did not occur, this was the first publication on the **bpp**–Co complex, and the early report was seminal in illustrating how **bpp** systematically alters electrochemical profiles.

Years later, in 2013, Burschka et al. [29] introduced a cobalt(III)–**bpp** complex known as FK269 (formulated as [Co(**bpp**)_2_]TFSI_3_ (Figure 7a), where TFSI is bis(trifluoromethyl-sulfonyl)imide) as a high-potential p-dopant for 2,*2′*,7,*7′*-Tetrakis[*N*,*N*-di(4-methoxyphenyl)amino]-9,9′-spirobifluorene (spiro-MeOTAD) in dye-sensitized solar cells (DSSCs). By virtue of the diamagnetic, LS Co(III) center, FK269 efficiently oxidized spiro-MeOTAD, enhancing hole transport and improving device performance. The switch to a TFSI counterion also boosted solubility. The novelty rested in achieving stronger doping capacity and better doping uniformity than previous Co-based dopants [18,34], an essential feature for stable solar cells.

Meanwhile, Świtlicka et al. turned to SIM in Co(II)–**bpp** complexes. In 2018 [32], they reported pentacoordinate Co(II) centers featuring **bpp** and halide (Cl^−^) or pseudohalide (NCS^−^, NCO^−^) ligands (Figure 7b). Despite being firmly HS and having a positive axial zero-field splitting D, the complexes displayed slow magnetic relaxation under applied fields, establishing that large positive D-values can enable field-induced SIM behavior even in Co(II) systems. Further work in 2020 [30] confirmed that hexacoordinate Co(II) centers with **bpp** (Figure 7a) and bridging pseudohalides also exhibit field-induced SIM, revealing multiple relaxation channels on the order of seconds at low temperatures.

In the same timeframe, Xiao et al. reported the reversible five-to-six-coordinate transformation of cobalt(II) complexes with **bpp**, highlighting their structural adaptability and dynamic behavior [33]. The five-coordinate complex [Co(**bpp**)Cl_2_] (Figure 7b) is initially isolated as a blue solid but rapidly absorbs moisture from the atmosphere, forming the pink six-coordinate species [Co(**bpp**)(H_2_O)_3_]Cl_2_. This transformation is fully reversible upon desiccation or solvation in acetonitrile. X-ray crystallography reveals that [Co(**bpp**)Cl_2_] adopts a distorted trigonal bipyramidal geometry, while [Co(**bpp**)(H_2_O)_3_]Cl_2_ assumes an octahedral coordination environment. Magnetic susceptibility measurements confirm a high-spin d^7^ Co(II) electronic configuration, with [Co(**bpp**)(H_2_O)_3_]Cl_2_ exhibiting a slightly higher magnetic moment (5.45 B.M.) due to additional orbital contributions. UV–visible spectroscopy shows a broad absorption at 677 nm for [Co(**bpp**)Cl_2_], characteristic of five-coordinate geometry, which shifts to 540 nm in [Co(**bpp**)(H_2_O)_3_]Cl_2_, consistent with octahedral coordination. This reversible transformation and its spectroscopic response suggest potential applications of **bpp**-based Co(II) complexes in stimuli-responsive materials and coordination-driven switching systems.

Cobalt–**bpp** complexes gained additional relevance for electrochemical energy storage thanks to Yang et al. in 2018 [31]. They discovered that a tridentate **bpp** environment [Co(II)(**bpp**)*_2_*]^2+^ (Figure 7a) can undergo SCO upon oxidation to Co(III). This redox-triggered SCO lowers the structural strain by distributing the added charge across the Co–N bonds, thereby stabilizing the oxidation state and ensuring high reversibility in the battery. They reported an ~88% capacity retention after 200 cycles, underscoring the synergy between the SCO process and stable electrochemical cycling. Notably, Co(**bpp**)*_2_* achieves a working potential of ~4.0 V vs. Li/Li^+^ in an RFB, among the highest reported for organometallic catholytes. The same group highlighted that the spin transition reduced molecular degradation, which is crucial in organic–inorganic redox flow systems operating at high voltages.

Related doping strategies were also extended to perovskite photovoltaics. In 2019, Lu et al. [28] used Co(II/III)(**bpp**)_2_ complexes (Figure 7a) to enhance hole transport in the spiro-MeOTAD layer and simultaneously protect the perovskite surface from harmful additives. The multivalent doping and interface stabilization functionality illustrate how these cobalt–**bpp** complexes can serve multiple roles in advanced solar cell architectures.

### 2.4. Ruthenium Complexes in Photovoltaic and Luminescent Applications

Chakrabarty et al. reported the synthesis and characterization of Ru(II) and Pd(II) complexes with **bpp**, namely [Ru(**bpp**)(DMSO)Cl_2_] and [Pd(**bpp**)Cl]Cl [35]. The Ru(II) complex exhibits a distorted octahedral geometry with **bpp** acting as a tridentate ligand, coordinating through two pyrazolyl atoms and one pyridine nitrogen atom. IR and UV–visible spectroscopic analyses confirmed the coordination mode, while cyclic voltammetry revealed two quasi-reversible redox processes attributed to the Ru^3+^/Ru^2+^ (E_1/2_ = 0.37 V) and Ru^4+^/Ru^3+^ (E_1/2_ = 1.0 V) couples. The electronic spectrum displayed d–d transitions (535–690 nm) and MLCT bands (405–430 nm), indicative of its electronic properties. The Pd(II) complex, [Pd(**bpp**)Cl]Cl, adopts a square planar geometry, with **bpp** coordinated in a tridentate manner. Its electronic spectrum showed a broad d–d transition (~400 nm) and intraligand π–π* transitions (250–300 nm), similar to the Ru complex. ^1^H NMR spectra confirmed ligand coordination, with characteristic shifts in the pyrazolyl and pyridine proton signals. These results highlight the distinct electronic and redox properties of Ru(II)–**bpp** and Pd(II)–**bpp** complexes, which may influence their applications in catalysis and optoelectronic materials.

Beyond their fundamental electronic and redox properties, Ru(II)–**bpp** complexes have also been studied for their potential in photoactive applications. For instance, in 2007, Philippopoulos et al. described five heteroleptic Ru(II) complexes bearing **bpp** and carboxyl-functionalized bipyridines (dcbpy) substituted at the 4 and 4′ positions of bipyridine (bpy): [Ru(**bpp**)Cl_3_], [Ru(**bpp**)(dcbpyH)Cl], [Ru(**bpp**)(dcbpyH_2_)Cl]Cl, [Ru(**bpp**)(dcbpyH)(NCS)], and Na[Ru(**bpp**)(dcbpy)(CN)] [36]. The aim was to explore these complexes as sensitizers in DSSCs, with dcbpy and its protonated variants (dcbpyH and dcbpyH_2_) providing anchoring groups for the TiO_2_ photoanode. Complex [Ru(**bpp**)Cl_3_] served as a key synthetic precursor, enabling successive ligand substitutions that introduced additional functional groups. These complexes showed MLCT absorption in the visible range and were tested as dyes in TiO_2_-based DSSCs. The broad absorption of complexes extending into the near-infrared (NIR) region and their electrochemical behavior, with reversible Ru(II)/Ru(III) redox couples, highlighted their promising potential for DSSC applications. When tested in photovoltaic cells, complexes with more carboxyl functional groups offered better adsorption on TiO_2_ and improved electron injection, leading to higher overall efficiencies. Specifically, [Ru(**bpp**)(dcbpyH_2_)Cl]Cl, bearing two protonated carboxyl groups (dcbpyH_2_), reached an efficiency of 1.12%, which, while lower than the benchmark N719 dye, still demonstrated the functional utility of these **bpp**-based complexes. Complexes [Ru(**bpp**)(dcbpyH)Cl] (with one carboxyl) and [Ru(**bpp**)(dcbpyH)(NCS)] (bearing an isothiocyanate ligand) yielded efficiencies of about 0.5%, while the cyanide-containing complex Na[Ru(**bpp**)(dcbpy)(CN)] with no carboxyl groups had the lowest performance under the reported conditions. Despite the modest numerical values, a positive outcome emerged in that the presence and positioning of carboxyl groups directly influenced the anchoring to TiO_2_, and the introduction of NCS^−^ to complex [Ru(**bpp**)(dcbpyH)(NCS)] fine-tuned the Ru t_2g_ orbitals in a way that stabilized certain electronic states, producing slightly higher performances than [Ru(**bpp**)(dcbpyH)Cl]. This work was the first report of heteroleptic Ru(II) complexes containing **bpp** ligands used as sensitizers in DSSCs, demonstrating the fundamental viability of **bpp**-based dyes in solar cell applications.

**Figure 8:**
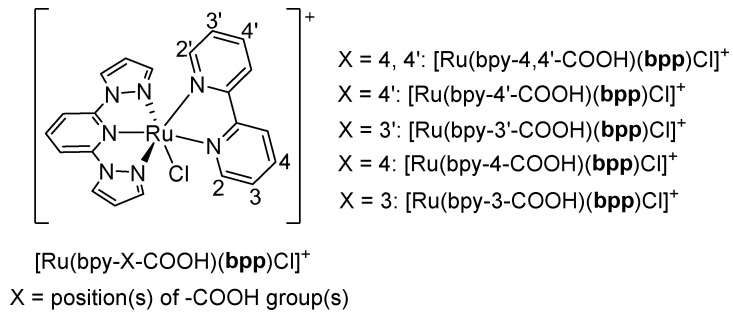
Structure of the Ru(II) complexes described in [37].

A subsequent contribution in 2018 by Lei et al. refined these concepts by examining the effect of the linkage position of carboxyl substituents in bpy on the performance of ruthenium dyes containing **bpp** ligands [37]. In that study, the authors synthesized a series of complexes generally formulated as [Ru(bpy-X-COOH)(**bpp**)Cl]^+^, where the position and number of -COOH groups on the bpy ring varied (Figure 8). Their goal was to elucidate how the regiochemistry of the anchoring group on bpy could optimize both light absorption and the current density in a DSSC. They first described [Ru(bpy-4,4′-COOH)(**bpp**)Cl]^+^, which bore -COOH groups at both the 4 and 4′ positions of bpy while retaining an unsubstituted **bpp** ligand. This complex showed strong absorption across 270–800 nm dominated by MLCT transitions and fulfilled the required HOMO–LUMO alignment for efficient electron injection. When applied to a DSSC, [Ru(bpy-4,4′-COOH)(**bpp**)Cl]^+^ yielded a short-circuit current density (J_sc_) of 13.15 mA/cm^2^, making it a strong reference standard in the study. In contrast, [Ru(bpy-4′-COOH)(**bpp**)Cl]^+^, containing only one -COOH group at the 4′ position of bpy, displayed a red-shifted absorption peak around 565 nm but decreased light-harvesting efficiency overall, leading to a lower J_sc_ of 9.53 mA/cm^2^. Similarly, [Ru(bpy-3′-COOH)(**bpp**)Cl]^+^ displayed a superstrong UV absorption by virtue of its 3′-COOH substitution pattern, although its resulting J_sc_ was only 10.26 mA/cm^2^. The biggest improvement appeared in [Ru(bpy-4-COOH)(**bpp**)Cl]^+^, which showed an additional absorption peak at 554 nm and a more effective utilization of the lower-energy region of the solar spectrum. This complex featured the highest reported J_sc_ of 13.48 mA/cm^2^ among the series, surpassing [Ru(bpy-4,4′-COOH)(**bpp**)Cl]^+^. This improvement was attributed to a more optimal distribution of electron density when the carboxyl substituent was placed at the 4 position of the bpy ring, enabling enhanced charge transfer and better solar energy coverage. A related analog, [Ru(bpy-3-COOH)(**bpp**)Cl]^+^, followed similar trends as [Ru(bpy-3′-COOH)(**bpp**)Cl]^+^, with a strong UV absorption but a lower J_sc_ value of 10.37 mA/cm^2^. Thus, a clear relationship emerged between carboxyl placement on bpy and the absorption characteristics, which translated directly into measurable differences in device performance. The authors highlighted the novelty of tuning electronic properties through careful positioning of carboxyl anchors. These structural differences modulate MLCT transitions and the alignment of the frontier orbitals, ultimately improving or diminishing photocurrent generation in a DSSC. The synergy that did emerge was between the fine-tuned absorption in the visible region and the optimized anchoring arrangement, leading to a better electron transfer and an enhanced photocurrent.

Both studies highlight the crucial role of ligand design in optimizing Ru(II) complexes for DSSCs. The work by Philippopoulos et al. demonstrated that **bpp**-containing complexes with carboxyl-functionalized bpy enhance TiO_2_ anchoring and electron injection, establishing their viability as sensitizers [36]. The study by Lei et al. [37] refined this approach, showing that the position of carboxyl groups on bpy significantly influences light absorption and photocurrent generation, with a single 4-carboxyl group yielding the best performance. Together, these studies emphasize the synergy between the ligand structure and electronic properties, guiding the design of more efficient DSSC sensitizers.

### 2.5. Complexes with Rhenium, Platinum, and f-Metals: Expanding the Photophysical Horizon

Rhenium(I) complexes of **bpp** were explored by Sangilipandi et al. in 2015 [38]. They synthesized fac-[Re(**bpp**)(CO)_3_Br] and demonstrated how the **bpp** ligand affords characteristic Re(I) MLCT absorption bands along with broad emission in the red-to-NIR region. DFT and TDDFT analyses confirmed that the HOMO is heavily localized on the Re–Br center while the LUMO resides on the ligand orbitals. The complex displayed robust luminescence and charge transfer bands important for potential photocatalysis or photosensitization.

In a similar vein, platinum(II) complexes with **bpp** have been investigated for their luminescent behavior, as reported by Willison et al. in 2004 [39]. They synthesized square planar [Pt(**bpp**)Cl]Cl·H_2_O and [Pt(**bpp**)(Ph)](PF_6_), observing distinct MLCT, chloride or phenyl ligand-to-metal charge transfer (LMCT), and chloride or phenyl ligand-to-ligand (**bpp**) charge transfer (LLCT) states. In the case of [Pt(**bpp**)(Ph)](PF_6_), which displays an intense and narrow emission around 655 nm at low temperatures, and assuming this emission originates from the lowest excited state, it was concluded that the lowest ligand field states must lie at or above the emission onset (≤16,900 cm^−1^). These findings provided a new path to near-red or red emitters that rely on the **bpp** scaffold.

Finally, a luminescent example with an f-metal emerged from the 2011 work of Cui et al. [40]. They prepared heterometallic coordination polymers [Pr**bpp**Ag_3_(SCN)_6_·H_2_O]_n_ and [Pr**bpp**Ag_3_(SCN)_6_]_n_, in which **bpp** participated in a sensitizing praseodymium(III)-based emission, leading to characteristic green-to-red transitions of Pr^3+^. This effect resulted from the “antenna effect” of **bpp** and the Pr ion’s luminescent excited states. The study spotlighted how the **bpp** framework can facilitate a strong metal-centered emission in lanthanide complexes by promoting energy transfer from the ligand’s π* orbitals.

In summary, the **bpp** ligand serves as a remarkably adaptable motif for constructing complexes that exhibit far more than simple coordination geometry changes. Its moderate ligand field strength can place 3d ions in borderline regimes conducive to SCO, especially for iron(II) or cobalt(II/III). The ability of **bpp** to support SCO has been synergistically combined with dielectric, electrical, optical, and redox properties. Iron(II) complexes have furnished spin state switching correlated with luminescence or electronic conduction, including the remarkable on/off electroluminescent devices of Matsuda and Tajima. Cobalt-based systems, on the other hand, have shown how redox-triggered SCO can stabilize higher oxidation states, improve redox reversibility, and even function as dopants in solar cells or as catholytes in high-voltage flow batteries. In parallel, **bpp**-based ruthenium, rhenium, platinum, and praseodymium complexes, while not displaying SCO, bring to light the ligand’s ability to tune MLCT, luminescent output, and framework topology. Across all these examples, whether the target is spin state switching, luminescence, or charge transport, the modular architecture of **bpp** continues to enable novel strategies for multifunctional coordination materials. The breadth of these applications—and the novelty found in each domain—reflects the enduring importance of the **bpp** ligand in contemporary coordination chemistry and materials science.

## 3. Functionalized bpp Ligands—Carboxylate and Ester Derivatives

**Figure 9:**
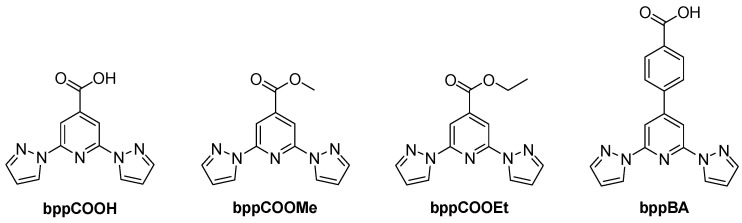
Structures and abbreviations of the ligands discussed in Section 3.

Building upon the fundamental properties of **bpp**, various functionalized derivatives have been developed to enhance its coordination ability, electronic properties, and structural versatility. Among these, carboxylate and ester-substituted derivatives—such as 2,6-bis(pyrazol-1-yl)isonicotinic acid (**bppCOOH**), 2,6-bis(pyrazol-1-yl)pyridine-4-carboxylate (**bppCOOMe**), its ethyl ester counterpart (**bppCOOEt**), and the benzoic acid-functionalized analog 4-(2,6-bis(1h-pyrazol-1-yl)pyridine-4-yl)benzoic acid (**bppBA**) were studied (Figure 9).

### 3.1. 2,6-Bis(pyrazol-1-yl)isonicotinic Acid (**bppCOOH**) and 2,6-Bis(pyrazol-1-yl)pyridine-4-ethylcarboxylate (**bppCOOEt**)

In 2009, Sivakumar et al. reported a heteroleptic ruthenium sensitizer incorporating a **bppCOOH** ligand, which was explored for DSSCs [41]. This ruthenium(II) complex, formulated as [Ru(**bppCOOH**)(dcbpyH_2_)Cl]Cl, combined **bppCOOH** and dcbpyH_2_ to enhance visible light absorption and anchoring on TiO_2_ surfaces. Spectroscopic measurements showed intense MLCT bands extending up to 610 nm, with an extinction coefficient of about 3000 M^−1^ cm^−1^, suitable for harvesting low-energy photons. Under standard dye-sensitized solar cell conditions, the complex reached a conversion efficiency of 1.9%, with a photocurrent density of 2.1 mA/cm^2^ and a photovoltage of 413 mV. Although the device performance did not rival benchmark ruthenium dyes such as N_3_, the work underscored how adding a carboxylate-functionalized tridentate ligand could stabilize the excited state and promote electron injection into the semiconductor. The complex functioned primarily as a photovoltaic dye, highlighting the role of **bpp**-type ligands (or **bppCOOH** in this instance) in tuning photoelectrochemical properties.

**Figure 10:**
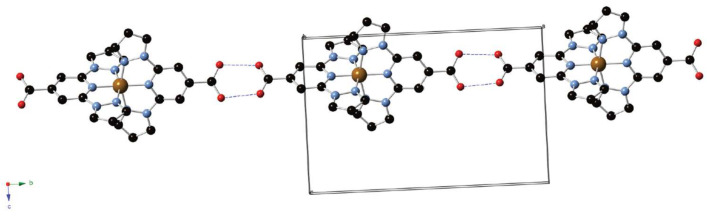
Supramolecular 1D hydrogen-bonded chain structure of the complex [Fe(II)(**bppCOOH**)_2_](ClO_4_)_2_. Color code: Fe(II) = brown, C = black, N = blue, O = red. The counterions and hydrogen atoms are omitted for clarity. Reprinted with permission from [42], Copyright (2014), Royal Society of Chemistry.

Subsequently, in 2014, Abhervé et al. were the first to introduce **bppCOOH** in an iron(II) SCO complex [42]. Their mononuclear compound [Fe(**bppCOOH**)_2_](ClO_4_)_2_ showed an abrupt spin transition near 384 K on heating and 381 K on cooling, revealing a narrow thermal hysteresis of about 3 K. This sharp SCO was traced to the strong hydrogen-bonded chains formed by the carboxyl groups, which effectively transmitted cooperative interactions among the metal centers (Figure 10). The authors also observed a remarkably high light-induced excited spin state trapping temperature (T_LIESST_) of 60 K, whereby light-induced conversion to the HS state could be photo-trapped down to temperatures significantly lower than is typical for similar **bpp**-based complexes. This work was particularly novel because it proved that attaching a carboxylic acid to the **bpp** core could yield a highly cooperative SCO material with exceptional photomagnetic properties. The carboxyl function additionally opened avenues for anchoring the system onto surfaces or for assembling more elaborate architectures.

**Figure 11:**
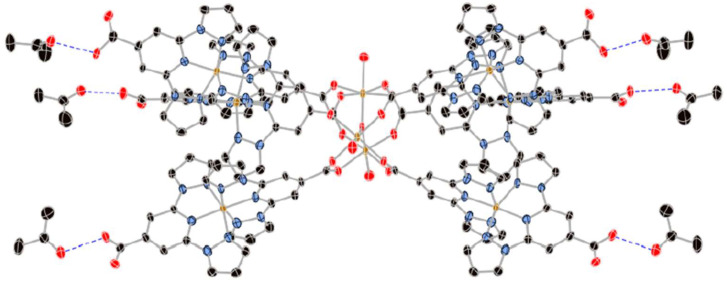
X-ray structure of the complex [Fe(III)_3_O(H_2_O)_3_(Fe(II)(**bppCOOH**)(**bppCOO**))_6_]^13+^ in the structure of [Fe(III)_3_(μ_3_-O)(H_2_O)_3_[Fe(II)(**bppCOOH**)(**bppCOO**)]_6_](ClO_4_)_13_·(CH_3_)_2_CO)_6_·(solvate) with acetone molecules forming hydrogen bonds (blue dashed lines). Color code: Fe = brown, C = black, N = blue, O =red. Hydrogen atoms were omitted for clarity. Reprinted with permission from [43], Copyright (2016), American Chemical Society.

In 2016, the same group expanded on the **bppCOOH** platform by constructing a nonanuclear cluster containing both Fe(II) and Fe(III) centers [43]. They synthesized a trigonal cluster with a central Fe(III)_3_(μ_3_-O) core and six peripheral [Fe(II)(**bppCOOH**)(**bppCOO^−^**)]^+^ units (Figure 11). While the Fe(III) sites exhibited antiferromagnetic coupling without SCO, the outer Fe(II) ions displayed a gradual and incomplete SCO in the 300–400 K range, reaching about 60% HS at 400 K. Because the Fe(II) centers lacked extensive intermolecular hydrogen-bonding networks, the transition was less cooperative than in the earlier mononuclear complex. The partial presence of both Fe(III) magnetism and Fe(II) SCO introduced a modest interplay, although the authors did not describe a strong synergy that might combine these properties into a single robust phenomenon. They did, however, emphasize the novelty of using the **bppCOO^−^** derivative as a bridging ligand, thereby demonstrating the feasibility of forming complex polynuclear and mixed-valence architectures with SCO-active components.

The spin state behavior of Fe(II) complexes based on the **bpp** ligand is intricately controlled by a combination of electronic and structural factors. Two complementary studies conducted in 2016 by Kershaw Cook et al. [44] and in 2024 by Berdiell et al. [45] provided detailed insights into how ligand substituents at the 4-position and angular distortions from ideal geometry influence the SCO transition temperature (T_1/2_) and the cooperativity of the transition. The study by Kershaw Cook et al. focused on the influence of electronic effects induced by substituents at the 4-position of the pyridine ring within the **bpp** framework [44]. Their work demonstrated that electron-withdrawing groups such as carboxyl (-COOH) significantly increase T_1/2_ by stabilizing the LS state. This effect is attributed to the inductive withdrawal of electron density from the pyridine nitrogen donor, which strengthens Fe(II) → ligand π-backbonding and increases ligand field splitting. As a result, the energy difference between the LS and HS states grows, leading to a higher T_1/2_. In contrast, the unsubstituted parent complex [Fe(**bpp**)_2_]^2+^ exhibits a lower T_1/2_, indicative of a moderate ligand field strength that allows a more thermally accessible SCO transition. This study further established a correlation between T_1/2_ and the Hammett parameters (σ_p_ and σ_p_^+^), revealing that the electronic nature of the substituents plays a decisive role in dictating the spin state equilibrium. DFT calculations confirmed that electron-withdrawing substituents primarily stabilize the t_2g_ orbitals of the Fe(II) center, reinforcing the ligand field strength and promoting an LS configuration. However, additional considerations such as ligand dissociation equilibria (observed for **bppCOOH**) may introduce secondary effects, although their impact on T_1/2_ in the solution appears minimal. While electronic effects provide a fundamental explanation for T_1/2_ variations, Berdiell et al. extended the discussion by examining the structural factors governing spin state transitions in [Fe(**bpp**)_2_]^2+^ complexes [45]. Their study combined crystallographic data and gas-phase DFT calculations to investigate angular deviations from ideal D_2d_ symmetry in HS states, revealing that distortions in the trans-N{pyridyl}–Fe–N{pyridyl} angle (ϕ) significantly affect the thermodynamic stability of the LS state. In the parent [Fe(**bpp**)_2_]^2+^ complex, ϕ frequently deviates below 160°, a distortion attributed to ligand flexibility and crystal packing effects. This deviation destabilizes the LS state, resulting in a less cooperative SCO process and an increased likelihood of kinetic trapping in the HS state. Interestingly, the study found that the introduction of electron-donating substituents (e.g., amino, sulfanyl, alkoxy) reduced the energy penalty associated with adopting distorted geometries. These groups promoted a stable distortion with ϕ ≈ 165°, a geometry that enhances cooperativity in the SCO transition. Conversely, electron-withdrawing substituents such as nitro and cyano had little effect on angular distortions, suggesting that their impact on T_1/2_ arises predominantly from electronic rather than steric considerations. Furthermore, these findings offer an explanation for why certain [Fe(**bpp**)_2_]^2+^ derivatives exhibit thermal hysteresis in their spin transitions. Complexes with rigid ligand environments that resist angular distortions tend to show incomplete spin transitions, while those with flexible, distortion-tolerant substituents are more likely to undergo cooperative SCO behavior. Both studies highlight that electron-withdrawing groups rigidify the ligand environment and favor the LS state, while electron-donating groups introduce greater structural flexibility, lowering T_1/2_ and promoting cooperative spin transitions. These findings collectively provide a robust framework for tuning SCO properties by balancing electronic effects with structural flexibility.

By 2018, several new reports showcased further properties of **bpp**-based ligands, including their SCO profiles, SIM, coordination polymers, and surface functionalization. One prominent study by the group of Clemente-León and Coronado illustrated how varying the counterion or the ligand substituent could systematically shift the SCO temperature [46]. They compared iron(II) complexes of **bppCOOH** and **bppCOOEt** with an array of anions (ClO_4_^−^, BF_4_^−^, CF_3_SO_3_^−^, AsF_6_^−^, SbF_6_^−^) to explore how anion size, lattice solvent, and hydrogen-bonding networks influenced the transition. For the smaller tetrahedral anions BF_4_^−^ and ClO_4_^−^, an abrupt and nearly complete SCO took place above about 340 K and 380 K, respectively, with a small thermal hysteresis of roughly 3 K. This cooperative behavior arose from the hydrogen-bonding chains formed by adjacent [Fe(**bppCOOH**)_2_]^2+^ units. In contrast, salts containing larger anions or lacking direct hydrogen bonding showed a more gradual SCO. Meanwhile, **bppCOOEt** complexes exhibited an SCO but were susceptible to irreversible desolvation when heated, implying that the carboxylic acid group in **bppCOOH** enhances reversibility. All the complexes showed notable tunability of the spin transition and photomagnetic light-induced excited spin state trapping (LIESST) properties. A parallel investigation that same year by the same group demonstrated SIM behavior in a mononuclear Co(II) complex with the **bppCOOH** ligand ([Co(II)(**bppCOOH**)_2_](ClO_4_)_2_·2Me_2_CO) [47]. This Co(II) center remained HS down to a low temperature and showed a slow relaxation of magnetization under an applied field, reflecting SIM properties. Moreover, when a small fraction of Fe(II) was replaced by Co(II) in the analogous SCO-active iron(II) lattice, the T_1/2_ shifted downward, and the cooperativity declined. Thus, rather than offering a beneficial synergy, doping with Co(II) diluted and broadened the iron(II) spin transition. While this compound did not display an SCO itself, it provided key insights into how mixed-metal approaches can modulate SCO parameters without necessarily producing a cooperative coupling of magnetic relaxation and spin transition.

Another 2018 study, already mentioned in Section 2, by Lei et al., also describes a complex of Ru(II), but this time the **bpp** ligand carries a single carboxyl (-COOH) group at the 4-position, while the bpy ligand remains unsubstituted, [Ru(bpy)(**bppCOOH**)Cl]^+^ [37]. This structural modification directs charge concentration toward the carboxyl group, optimizing electron injection into the semiconductor. Compared to [Ru(bpy-4-COOH)(**bpp**)Cl]^+^, which features a -COOH group on bpy and exhibits enhanced low-energy absorption, [Ru(bpy)(**bppCOOH**)Cl]^+^ has fewer low-energy absorption peaks but still maintains an efficient charge transfer. Its J_sc_ of 10.81 mA/cm^2^, slightly lower than that of [Ru(bpy-4-COOH)(**bpp**)Cl]^+^, demonstrates that **bpp**-based functionalization can effectively tune charge distribution in DSSCs. The study underscores how the placement of anchoring groups influences MLCT transitions and photovoltaic performance, with [Ru(bpy)(**bppCOOH**)Cl]^+^ emphasizing the role of **bpp** modifications in directing charge injection. While [Ru(bpy-4-COOH)(**bpp**)Cl]^+^ remains the most efficient design in this series, [Ru(bpy)(**bppCOOH**)Cl]^+^ presents an alternative approach to optimizing ligand-based charge transfer pathways in DSSC sensitizers.

**Figure 12:**
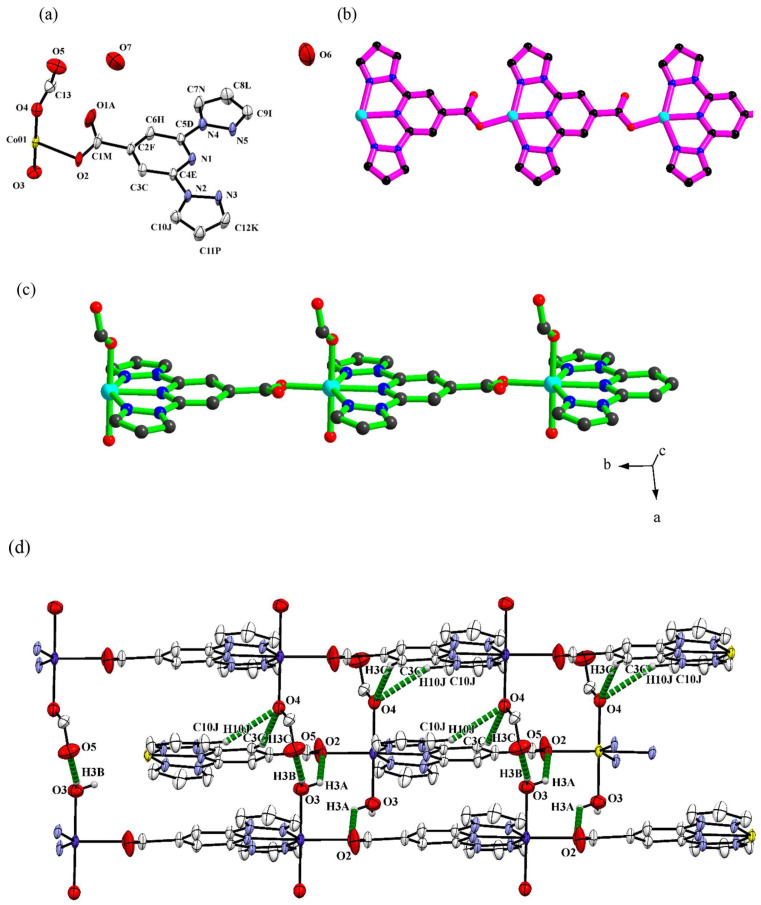
(**a**) Thermal ellipsoidal (50% probability level) plot of the asymmetric unit in the crystal structure of {Co(**bppCOO**)(HCOO)(H_2_O)}_n_·n1.5H_2_O. Color code: Co = yellow, C = light gray, N = blue, O = red. Hydrogen atoms are omitted for clarity. (**b**) Coordination of the **bppCOO**^−^ ligand with the Co(II) ion. (**c**) Connectivity of the **bppCOO**^−^ ligand with the cobalt(II) ion, water oxygen, and the formate ion, forming a chainlike structure in the crystal structure of {Co(**bppCOO**)(HCOO)(H_2_O)}_n_·n1·5H_2_O. (**d**) Three-dimensional supramolecular network formed by the intermolecular hydrogen bonding interactions in the crystal structure of compound {Co(**bppCOO**)(HCOO)(H_2_O)}_n_·n1.5H_2_O. Reprinted with permission from [48], Copyright (2019), American Chemical Society.

A separate publication in 2019 by Bommakanti et al. used **bppCOOH** in the construction of functional coordination polymers containing Zn, Cu, or Co, with an emphasis on single-crystal-to-single-crystal transformations and catalytic activity [48]. These coordination polymers showed notable behaviors. For instance, the Zn(II) polymer {Zn(**bppCOO**)_2_}_n_ served as a “host” lattice for complete transmetalation to produce {Cu(**bppCOO**)_2_}_n_, retaining crystallinity throughout the metal exchange. The Co(II) polymer {Co(**bppCOO**)(HCOO)(H_2_O)}_n_·n1·5H_2_O (Figure 12) displayed a preliminary electrocatalytic activity for water oxidation by virtue of the presence of Co–OH_2_ moieties accessible for higher-valent cobalt–oxo species formation. These outcomes underscored how the bifunctional character of **bppCOOH** (N-donor and carboxylate) could be harnessed in extended networks to achieve structural transformations or catalytic functionality.

**Figure 13:**
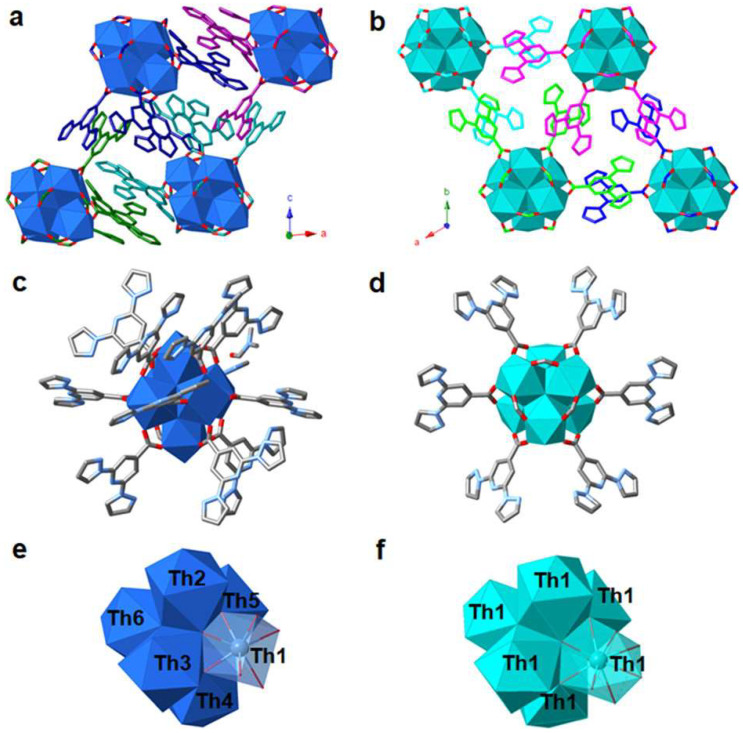
(**a**) Representation showing the packing of 0D clusters in Th-**bppCOO**-1. (**b**) Representation showing the packing of 0D clusters in Th-**bppCOO**-2. (**c**) Molecular structure of a single cluster of Th-**bppCOO**-1. (**d**) Molecular structure of a single cluster of Th-**bppCOO**-2. (**e**) Side view showing the [Th_6_(OH)_4_O_4_(H_2_O)_5_]^12+^ node in Th-**bppCOO**-1. (**f**) Side view showing the [Th_6_(OH)_4_O_4_(H_2_O)_6_]^12+^ node in Th-**bppCOO**-2. Color code: Th^4+^ polyhedra = dark blue or green, C = gray, N = light blue, O = red. Reprinted with permission from [49], Copyright (2021), American Chemical Society.

In 2021, the group of Lin reported the design and synthesis of two novel hexanuclear thorium clusters—Th-**bppCOO**-1 and Th-**bppCOO**-2—that integrate **bppCOOH** ligands into their structures, thereby establishing a new platform for selective X-ray dosimetry [49]. Th-**bppCOO**-1 features an unprecedented secondary building unit (SBU) of [Th_6_(OH)_4_O_4_(H_2_O)_5_]^12+^, in which only five water molecules coordinate to the Th_6_ core, in contrast to the typical [Th_6_(OH)_4_O_4_(H_2_O)_6_]^12+^ SBU found in Th-**bppCOO**-2. Single-crystal X-ray diffraction revealed that Th-**bppCOO**-1 crystallizes in the triclinic *P*1 space group while Th-**bppCOO**-2 adopts the trigonal *R*3 space group (Figure 13); notably, the closest interlamellar distance in Th-**bppCOO**-1 is 3.150 Å compared to 3.408 Å in Th-**bppCOO**-2, indicating distinct packing modes driven by subtle structural differences. Both complexes exhibit blue photoluminescence centered at 395 nm, with photoluminescence (PL) quantum yields of 10.26% for Th-**bppCOO**-1 and 14.32% for Th-**bppCOO**-2 and remarkably long lifetimes of 446.31 μs and 590.65 μs, respectively—far exceeding the 4.71 μs lifetime of the free ligands, an enhancement attributed to the heavy atom-induced intersystem crossing. Under X-ray irradiation (Cu Kα, 120 Gy/min), these clusters display a selective PL quenching response: the emission intensity of Th-**bppCOO**-2 drops to 49.1% at a dose of 0.01 kGy and further to 91.2% at 4.8 kGy, whereas Th-**bppCOO**-1 shows only 0.7% quenching at 0.01 kGy and 45.5% at 4.8 kGy; in stark contrast, exposure to UV light (λ_ex_ = 254 nm) causes negligible PL quenching. Complementary analyses including powder X-ray diffraction (PXRD), Fourier-transform infrared spectroscopy (FTIR), and electron paramagnetic resonance (EPR) (with post-irradiation g factors of 2.0085 for Th-**bppCOO**-1 and 2.0060 for Th-**bppCOO**-2) confirm that luminescence quenching arises from an X-ray-induced radical mechanism—specifically, the ionization of the **bppCOO^−^** ligands leading to the formation of stabilized **bppCOO^•^** radicals that enhance nonradiative decay—without any structural degradation, thereby underscoring the robustness of these materials for X-ray dosimetry applications over a wide dynamic range from Gray to kiloGray levels. This work on thorium-based clusters sheds light on the design and synthesis of actinide-bearing materials as novel sensors for ionizing radiation detection.

**Figure 14:**
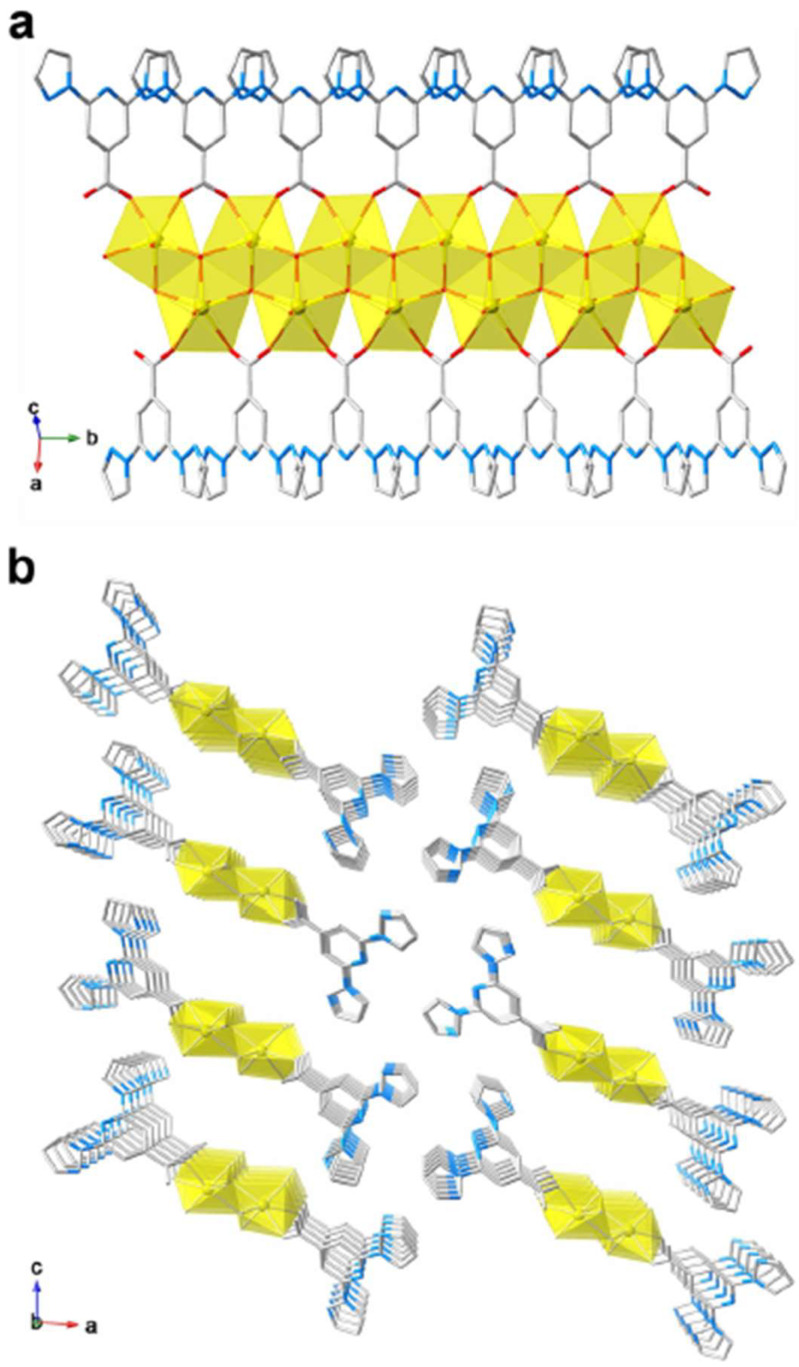
(**a**) A 1D chain of U–**bppCOO** showing the coordination environment of uranyl cations. (**b**) Polyhedral representation showing the 1D chain structure of U–**bppCOO** extending along the b-axis. Color code: U = yellow, C = gray, N = blue, O = red. Only one part of the two split sites of U and O atoms is shown for clarity. Reprinted with permission from [50], Copyright (2022), Royal Society of Chemistry.

In the same timeframe, in a study published in 2022, the same group showcased a uranyl coordination polymer U–**bppCOO** built on **bppCOOH** [50], conceptually aligning with the broad trend of functional materials from **bpp** derivatives. This one-dimensional polymer of UO_2_^2+^ and **bppCOO^−^** (Figure 14) exhibited enhanced photosensitivity to both UV and X-ray irradiation, reflected in pronounced luminescence quenching at low irradiation doses. The synergy here revolved around LMCT and high X-ray attenuation by the heavy uranyl cations, leading to dose-dependent quenching with a detection limit as low as 0.012 Gy for X-rays. The material’s photophysical response and luminescence sensitivity underscored the versatility of **bppCOOH** in assembling complexes with emergent optical properties.

### 3.2. 2,6-Bis(pyrazol-1-yl)pyridine-4-carboxylate (**bppCOOMe**)

Another noteworthy 2018 contribution by Rigamonti et al. highlighted a pseudo-octahedral cobalt(II) complex featuring methyl-carboxylate-functionalized **bpp** ligand, **bppCOOMe** [51]. Here, Co(II) remained HS and did not cross over, but the complex displayed robust SMM characteristics, including zero-field slow relaxation of magnetization when diluted in a diamagnetic Zn(II) lattice. The geometry imposed by two meridional **bppCOOMe** ligands fostered strong axial anisotropy, enabling magnetically bistable behavior. The work was distinctive in providing the first fully characterized example of a pseudo-octahedral HS Co(II) complex with zero-field SMM behavior—a breakthrough in designing cobalt-based molecular magnets outside of the usual distorted-tetrahedral motif.

### 3.3. 4-(2,6-Bis(1H-pyrazol-1-yl)pyridine-4-yl)benzoic Acid (**bppBA**)

Finally, the self-assembly potential of **bppBA** was explored by Aitchison et al., also in 2018 [52]. This study did not involve SCO metals but focused on forming self-assembled monolayers (SAMs) of **bppBA** on ultrathin silver deposits. Surface analyses revealed a highly ordered arrangement with a herringbone packing motif. The carboxylate group anchored the molecule to the silver underlayer, while the **bpp** ring systems stood upright, leading to a tightly packed and crystalline-ordered monolayer. The authors noted that, although the **bpp** moiety is capable of binding metals under other conditions, the packing density in this SAM left little space for further coordination at the pyrazole and pyridine sites. Such surface functionalization strategies, however, could pave the way for subsequent layer-by-layer construction of functional architectures, including those with potential SCO centers if carefully designed. The novelty lay in demonstrating that Ag-based interfaces allow strong carboxylate linkage alongside distinctive molecular ordering, providing a platform for advanced nanoscale functionalization.

Taken together, these studies illustrate how modifications of the **bpp** framework, whether through carboxylates or esters, yield an extensive family of coordination compounds and materials with diverse properties. The team of Coronado has extensively demonstrated how carboxyl and ester functionalization at the 4-position of **bpp** can fine-tune SCO properties, enhance cooperativity, and enable surface functionalization. Their research has spanned from mononuclear SCO-active Fe(II) complexes with sharp thermal transitions to polynuclear architectures that introduce mixed-valence effects. Through systematic modifications of the ligand environment—including counterion exchange, hydrogen-bonding networks, and metal substitutions—they have revealed how small structural changes dictate abruptness, reversibility, and LIESST properties. Their work with **bppCOOH** highlighted the role of hydrogen bonding in promoting cooperative SCO, whereas **bppCOOEt** provided insights into how ligand rigidity and solvation impact stability and reversibility. Building on the contributions of other research teams, additional carboxylate- and ester-substituted derivatives of **bpp** demonstrate catalytic water oxidation, photosensitivity to radiation, or photoelectrochemical performance when the ligand is partnered with uranyl or ruthenium for luminescent or solar-energy applications. Finally, substituting the carboxyl group onto the benzoic or isonicotinic ring permits robust surface anchoring, enabling self-assembly into monolayers or infiltration into semiconductor electrodes. The collective novelty stems from how these functional groups and coordination motifs can be selectively combined to produce materials that—depending on design—exhibit SCO transitions, magnetic bistability, catalytic reactivity, or tunable photophysical responses. This versatility underscores the importance of continuing to explore **bpp**-type ligands as a cornerstone for multifunctional coordination chemistry.

## 4. Aromatic Modifications of bpp Ligands

This chapter explores how aromatic substitutions at the 4-position of **bpp** ligands can profoundly influence the behavior of their metal complexes. We begin by examining pyrene-functionalized derivatives, where variations in linker rigidity—ranging from direct C–C bonds to more flexible spacers—demonstrate how even slight changes can dictate SCO properties in Fe(II) systems. The discussion then broadens to include fullerene-appended, radical-bearing, and helicene-based modifications, as well as modifications with aggregation-induced emission (AIE) activity. Each aromatic substituent alters key interactions, such as ligand field strength and π–π stacking, which, in turn, modulate electronic, photophysical, and magnetic characteristics. This comparative analysis underscores not only the versatile role of **bpp** as a platform for tuning complex properties, but also its potential for applications in spintronics, molecular electronics, and photonics.

### 4.1. Pyrene-Functionalized **bpp** Ligands and Their Complexes

**Figure 15:**
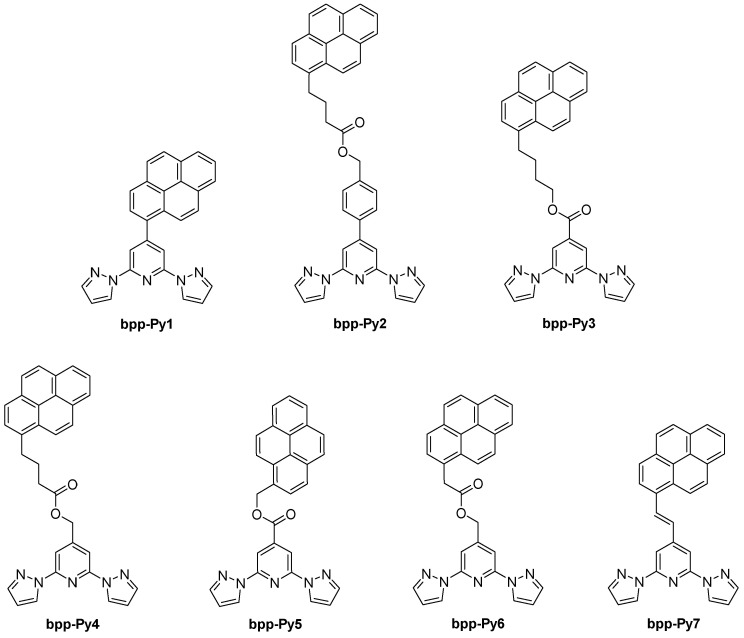
Structures and abbreviations of the pyrene-containing ligands discussed in Section 4.

The introduction of pyrene units onto the **bpp** framework (Figure 15) has proven especially popular for creating SCO complexes designed for potential electronic or photonic applications. Early on, in 2011, González-Prieto et al. explored how the structural flexibility around the pyrene substituent could tune the Fe(II) spin state in the solid state [53]. They synthesized two major ligand variants: one with a direct C–C linker between **bpp** and pyrene (**bpp-Py1**) and another incorporating a flexible butanoate–benzyl chain (**bpp-Py2**). Upon coordinating Fe(II), the first complex, [Fe(**bpp-Py1**)_2_](ClO_4_)_2_, remained HS across the entire temperature range because the rigid π–π stacking of pyrene moieties prevented the FeN_6_ core from contracting. By contrast, the Fe(II) complexes derived from the more flexible **bpp-Py2** underwent reversible SCO with T_1/2_ values around 216–218 K. The presence of pyrene also enabled fluorescence in solution, but temperature-dependent luminescence measurements showed no direct synergy with the SCO transition. This study illustrated that the spacer length and steric arrangement around the pyrene group are key to stabilizing either the LS or HS forms and to preserving SCO in the solid state.

Subsequent research by Kumar et al. in 2017 demonstrated that these same Fe(II)–**bpp**–pyrene complexes could be noncovalently anchored to graphene to produce a hybrid SCO-active material [54]. They deposited the [Fe(**bpp-Py2**)_2_](BF_4_)_2_ species onto exfoliated graphene sheets, confirming that the SCO remained intact, though slightly broadened and shifted to a higher T_1/2_ (232–242 K) relative to the bulk. This retention of spin-switching capacity on a 2D conductive substrate signaled the feasibility of integrating SCO molecules into future spintronic devices, although no direct conductivity modulation by the SCO was demonstrated.

By 2018, Burzurí et al. demonstrated that a single [Fe(**bpp-Py2**)_2_](BF_4_)_2_·CH_3_CN·H_2_O molecule could indeed mediate spin state-dependent conductance when anchored between graphene electrodes at the nanoscale [55]. Their low-temperature measurements revealed spontaneous telegraph-like switching between two conductance states that correlated with HS vs. LS configurations of the Fe center. DFT calculations supported the conclusion that even small structural fluctuations in a single molecule could trigger the LS⇄HS crossover, causing distinct conductance changes. This was the first demonstration of a direct SCO-driven electrical switching in an Fe(II)–**bpp**–pyrene single-molecule device, providing a vivid synergy between molecular spin state and electronic transport. As a result, these molecules have potential as dynamic spin polarizers, capable of being toggled based on their spin state.

In 2017, the group of Ruben expanded on the concept of linking pyrene anchors to **bpp**-based Fe(II) centers with systematically varied spacer lengths [56]. By adjusting –(CH_2_)_4_OOC– or –(CH_2_)_3_COOCH_2_– linkers at the 4-position of the **bpp** core, they achieved Fe(II) complexes whose T_1/2_ values spanned a wide range, from ~213 K up to 353 K. One example, [Fe(**bpp-Py3**)_2_](BF_4_)_2_, displayed a high T_1/2_ near 353 K with a small thermal hysteresis (~2 K), whereas another, [Fe(**bpp-Py4**)]_2_(BF_4_)_2_, switched at 213 K. According to the authors, the pronounced difference in T_1/2_ values between these two complexes arose from the distinct ligand field strengths exerted by **bpp-Py3** and **bpp-Py4**. In **bpp-Py3**, the presence of an electron-withdrawing carboxylic ester group at the pyridine 4-position lowers the π* orbitals on the **bpp** core and reinforces Fe–**bpp** π-backbonding, thereby stabilizing the LS state and elevating T_1/2_. By contrast, **bpp-Py4** includes a –CH_2_– substituent directly at the **bpp** ring, which reduces this orbital stabilization and thus lowers the T_1/2_ relative to **bpp-Py3**. Moreover, the fact that electronic effects prevail over steric considerations in this pyrene-decorated system supports the broader feasibility of developing single-molecule spintronic devices using functionalized Fe(II) SCO complexes. Both complexes also underwent photoinduced LS→HS conversion via the LIESST effect, confirming robust SCO that could be tuned by simple changes in the spacer.

A related 2018 study by the same group examined three Fe(II) complexes designed for supramolecular interactions [57]. The study featured ligands with short-linker pyrene derivatives, including one with a –COOCH_2_– spacer linking the **bpp** ligand to the pyrene group (**bpp-Py5**), and a similar derivative with a –CH_2_OR– spacer (**bpp-Py6**). The results demonstrated how these structural variations significantly influenced the SCO behaviors of the complexes. Notably, [Fe(**bpp-Py5**)_2_](BF_4_)_2_ exhibited a high-temperature, gradual SCO with T_1/2_ ≈ 450 K; at 450 K, about 70% of the molecules are in the HS state (χT ≈ 2.41 cm^3^·K·mol^−1^). Photomagnetic measurements at 10 K (LS) under 637 nm irradiation yielded up to ~80% HS fraction, with the metastable HS state relaxing at T_LIESST_ = 40 K. In contrast, [Fe(**bpp-Py6**)_2_](BF_4_)_2_ remained fully HS across the 300–5 K range (χT ≈ 3.40 cm^3^·K·mol^−1^ at 300 K), showing no thermal SCO. These results underscore the critical role of short spacers in restricting the lattice flexibility needed for a complete spin state transition.

### 4.2. Radical-Appended **bpp** Derivatives

**Figure 16:**
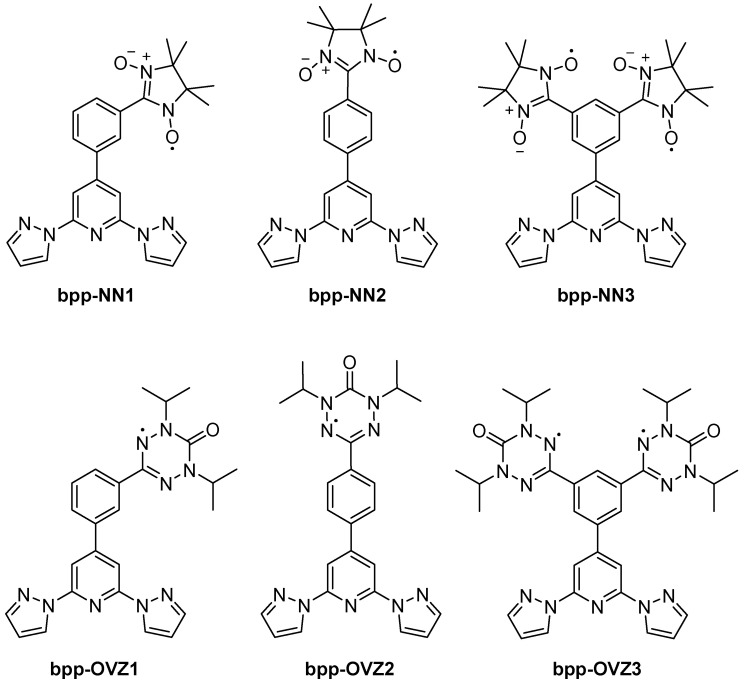
Structures and abbreviations of the radical-appended **bpp** derivatives discussed in Section 4.2.

Moving from hybrid SCO systems to purely organic radicals, in 2012, Hui et al. investigated **bpp** derivatives bearing nitronyl–nitroxide (NN) or oxoverdazyl (OVZ) functionalities (Figure 16) [58]. They focused on mono- and biradicals in which the **bpp** segment acts as a conjugated “coupling unit” that fixes the relative orientation of the two radical centers in the biradical derivatives, enabling systematic comparisons of the intramolecular magnetic interactions (zero-field splitting, hyperfine coupling, spin–spin exchange). Electron spin resonance (ESR) measurements confirm that the NN biradical has a larger dipolar coupling (zero-field splitting) than the OVZ biradical, indicating stronger spin delocalization across the **bpp**–phenyl backbone. This approach illustrated that the conjugated **bpp** bridge could modulate spin–spin communication between radical sites, offering a new molecular platform for magnetically active materials.

In a subsequent 2012 report, the same group showed that a **bpp-NN1** radical could self-assemble into various nano- and microstructures, such as vesicles, porous films, and nanoparticles, depending on solvent conditions and ultrasound treatment [59]. EPR data confirmed retention of the S = 1/2 radical center, and remarkably, the crystalline nanoparticles could revert to vesicles over time, signifying a reversible morphological switch. While these studies did not coordinate metals to the radical-functionalized **bpp**, they emphasized how the **bpp** scaffold fosters both supramolecular organization and robust paramagnetism.

### 4.3. Phosphazene-Linked Ruthenium Complexes

**Figure 17:**
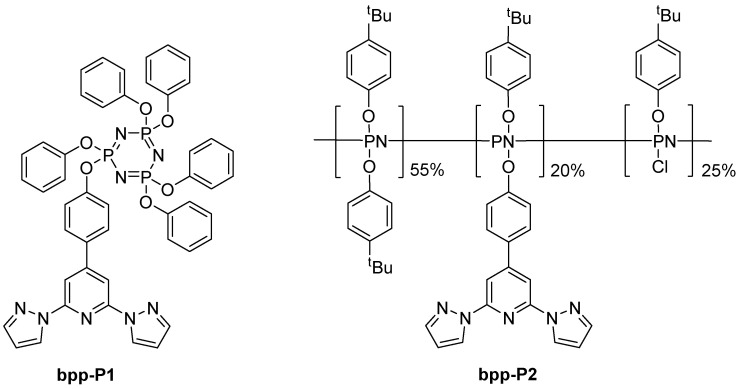
Structures and abbreviations of the phosphazene-containing ligands discussed in Section 4.3.

A different branch of research appeared in 2015, when Davidson et al. reported a series of cyclotriphosphazene and polyphosphazene ruthenium(II) compounds using 2,2′:6′,2″-terpyridine (terpy) and **bp**p units (Figure 17) [60]. A distinctive set of properties sets **bpp**-containing derivatives apart from their bis-terpy counterparts. Their electronic spectra reveal a marked blue shift, with MLCT bands appearing in the 432–440 nm region, in contrast to the ~490 nm bands typical of the bis-terpy complexes. Additionally, these **bpp**–terpy complexes feature a secondary absorption band between 350 and 380 nm—more prominent in complexes [Ru(**bpp-P1**)(terpy)](PF_6_)_2_ and [Ru(**bpp-P1**)(Phterpy)](PF_6_)_2_—which indicates that the MLCT transitions involve a delocalized interplay between the **bpp** and terpy moieties, as supported by DFT calculations. Resonance Raman measurements further reinforce this observation by displaying delocalized vibrational modes below 700 cm^−1^, suggesting a similar chromophore environment across the small-molecule **bpp** derivatives. In terms of electrochemistry, although **bpp** is inherently more electron-donating than terpy, the oxidation potentials of these complexes do not follow a straightforward trend; subtle shifts are observed, likely due to conformational changes upon oxidation and the complex interactions between **bpp** and the co-ligands. Finally, when these derivatives are incorporated into polymeric systems, specifically in [Ru(**bpp-P2**)(terpy)]Cl_2_] and [Ru(**bpp-P2**)(Phterpy)]Cl_2_], their absorbance profiles become broader and less defined, hinting at an altered coordination environment, potentially stemming from the milder reaction conditions and incomplete anion exchange during polymer synthesis. Overall, these findings illustrate how the integration of the **bpp** moiety fine-tunes both the optical and redox properties of ruthenium complexes, albeit with added complexity when extended to polymeric frameworks.

### 4.4. Fullerene-Appended **bpp** Systems

**Figure 18:**
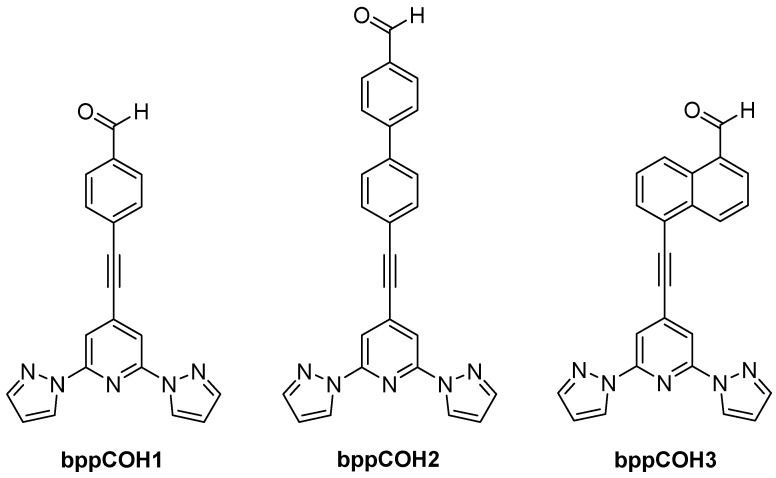
Structures and abbreviations of the ligands described in [61].

Fullerene anchoring to **bpp**-based complexes is another strategy for constructing molecular spin-electronic devices. In 2017, Nuin et al. introduced a synthetic approach in which Fe(II) or Co(II) complexes with ligands (depicted in Figure 18) were appended to C_60_ via the Prato reaction [61]. Their work primarily focused on optimizing yields and structural characterization. The novelty lay in showing that fullerene end-caps could serve as robust anchoring moieties for prospective single-molecule junctions, with **bpp** ensuring an octahedral coordination environment for first-row transition metal centers.

**Figure 19:**
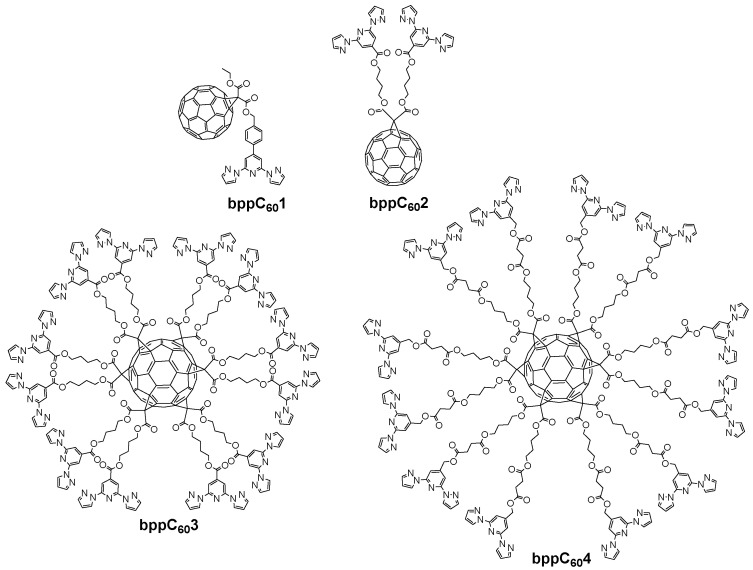
Structures and abbreviations of the fullerene-appended **bpp** systems discussed in Section 4.

Subsequent studies by Kumar et al. in 2018 expanded on attaching both pyrene and fullerene to a single **bpp**–Fe(II) complex to examine their influence on SCO behavior [57]. These large pyrene π-systems led to partial or very gradual spin transitions, as noted earlier in Section 4.1. Additionally, they investigated a complex featuring a bulky fullerene (C_60_) substituent in the ligand **bppC_60_1** (Figure 19). The fullerene-tethered [Fe(**bppC_60_1**)_2_](BF_4_)_2_ started in the HS state at 385 K (molar magnetic susceptibility–temperature product, χT ≈ 3.13 cm^3^·K·mol^−1^). Upon cooling, it underwent a gradual but incomplete SCO, centered at 208 K, achieving only ~50% conversion to LS below 100 K. Three years later, the group of Coronado reported a more elaborate design based on mono-/hexakis-adducts of C_60_ bearing multiple **bpp** arms (**bppC_60_2, bppC_60_3,** and **bppC_60_4** depicted in Figure 19) [62]. By introducing up to twelve **bpp** sites onto a single C_60_, they could coordinate as many as six Fe(II) centers around one fullerene core. Thermally induced SCO was observed in these polynuclear clusters, albeit in a gradual and sometimes incomplete manner, and partial LIESST was achieved at low temperatures. This work highlighted how fullerene’s three-dimensional scaffold can promote cooperative interactions among multiple Fe(II) sites, thereby enhancing collective SCO behavior compared to mononuclear complexes.

### 4.5. Helicene-Based **bpp** Derivatives

**Figure 20:**
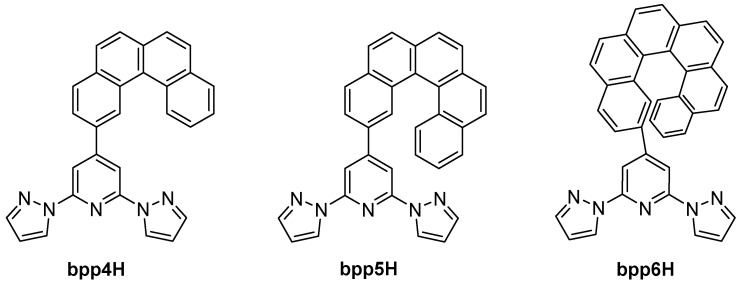
Structures and abbreviations of the helicene-based **bpp** ligands.

Along a photophysical trajectory, in 2019, the group of Avarvari explored **bpp** ligands bearing helicene substituents at the 4-position of the pyridine ring [63]. Their report covered both Ru(II) and Re(I) complexes containing **bpp[n]H** helicenes, where n = 4, 5, 6 (Figure 20). The Ru(II) complexes exhibited predominantly ^3^MLCT phosphorescence at 77 K, whereas the Re(I) complexes displayed intense emission in the visible region at low temperatures, likely from a ligand-localized π–π* phosphorescence. The helicene moiety conferred chirality, and racemic crystals were isolated. The strong luminescent response in d^6^ Ru or d^5^ Re systems and helicene-driven chirality pointed to potential applications in circularly polarized luminescence (CPL).

In 2022, the same group obtained enantiopure **bpp6H** ligands and coordinated them to Eu(III) and Yb(III) centers [64]. The Eu(III) complexes emitted in the red region with CPL, achieving glum values on the order of 10^−3^, while the Yb(III) complexes luminesced in the NIR region with no observable CPL. These examples revealed that the **bpp**–helicene platform can sensitize lanthanide emission and impart chiroptical activity, though the magnitude of CPL remained limited by the spatial separation between the helicene unit and the lanthanide center.

### 4.6. AIEgen- and Other Fluorescently Functionalized **bpp** Complexes

**Figure 21:**
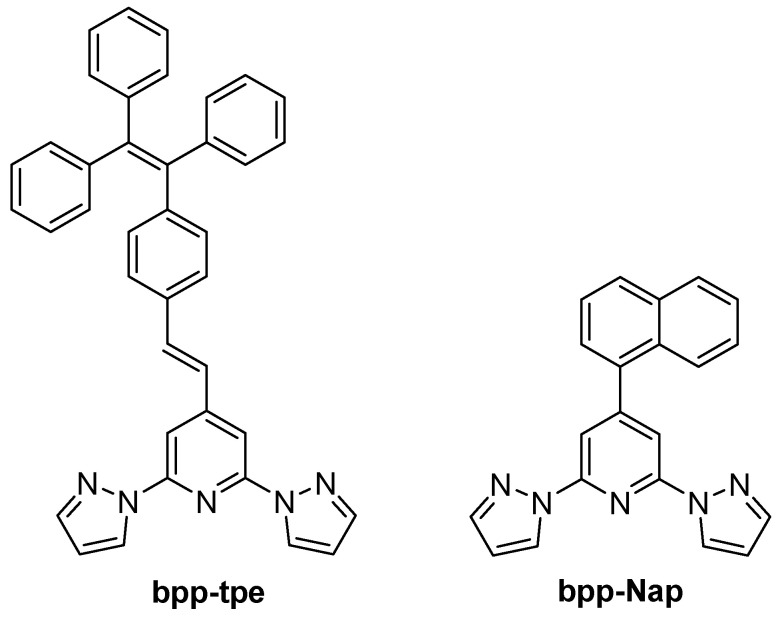
Structures of the tetraphenylethylene-based **bpp** ligand (**bpp-tpe**) [65,66] and the naphthalene-based **bpp** ligand (**bpp-Nap**) [67].

Most recently, research has shifted toward integrating AIE motifs into **bpp**-based transition metal complexes. In 2023, Yi et al. prepared three new Fe(II) mononuclear compounds bearing a tetraphenylethylene (tpe) moiety attached to a **bpp** core (**bpp-tpe**) (Figure 21) [65]. These complexes, [Fe(**bpp-tpe**)_2_](ClO_4_)_2_·5.75CH_2_Cl_2_, [Fe(**bpp-tpe**)_2_](ClO_4_)_2_·CH_2_Cl_2_·3CH_3_OH, and [Fe(**bpp-tpe**)]_2_(BF_4_)_2_·CH_2_Cl_2_·3CH_3_OH, each displayed temperature-induced SCO with T_1/2_ around 375 K, 260 K, and 248 K, respectively. All three were strongly emissive in the solid state due to the tpe-based AIE effect. However, the fluorescence intensity showed no abrupt response at the T_1/2_, implying little direct SCO–emission coupling. The authors concluded that the large spatial separation between tpe and the Fe(II) center prevents efficient energy transfer, and that minimal distortion in the tpe environment during SCO further hinders a spin state-driven luminescence switch. Nevertheless, they demonstrated that Fe(II)–**bpp** can accommodate a classic AIEgen without quenching its emission.

**Figure 22:**
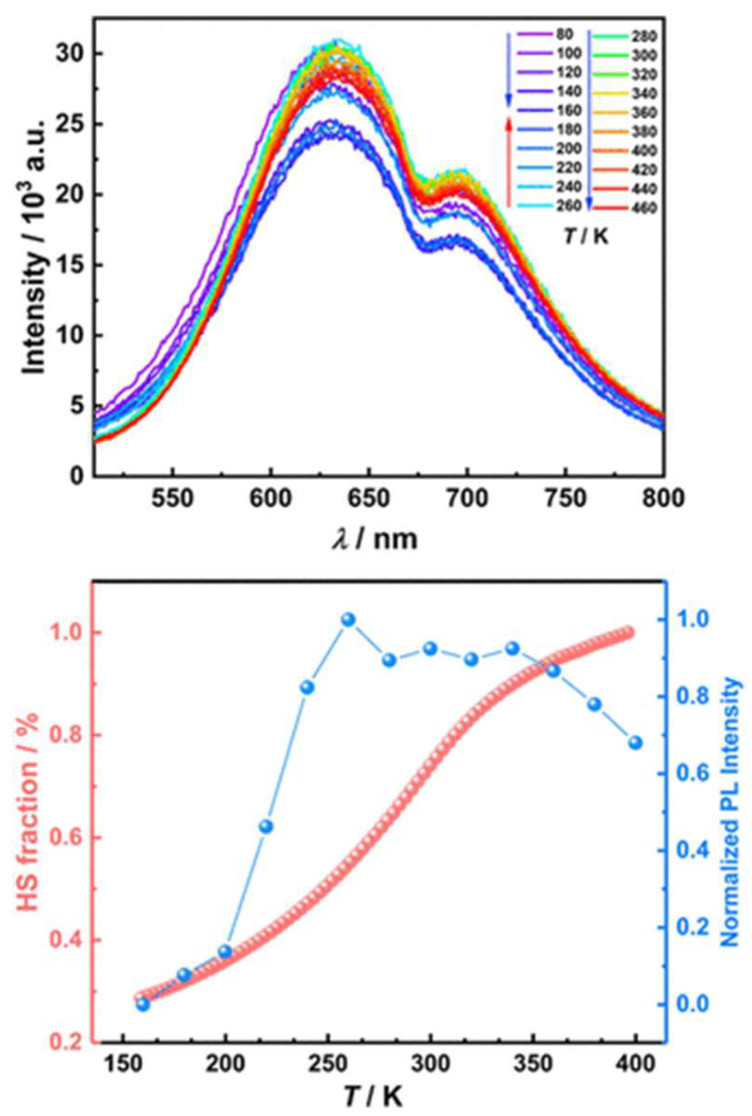
Temperature-dependent luminescent emission spectra for the desolvated sample of complex [Fe(bpp-tpe)_2_]^2+^ in the solid state, with λ_ex_ = 467 nm. The arrows indicate the trend of emission intensity with increasing temperature (**top**). Plots of the HS fraction of the Fe(II) ion (red) and photoluminescence intensity at 630 nm (blue) as a function of temperature for the desolvated sample of [Fe(bpp-tpe)_2_]^2+^ (**bottom**). Reprinted with permission from [66], Copyright (2025), American Chemical Society.

Expanding upon this trend, in 2025б Wu et al. synthesized and characterized a mononuclear Fe(II) complex [Fe(bpp-tpe)_2_](ClO_4_)_2_·H_2_O·0.5CH_2_Cl_2_ incorporating an AIE-active ligand (Figure 21) into an SCO-active Fe(II) coordination environment [66]. Unlike previous systems [65] where SCO and fluorescence remained largely independent due to the spatial separation between Fe(II) and the emissive units, [Fe(bpp-tpe)_2_](ClO_4_)_2_·H_2_O·0.5CH_2_Cl_2_ exhibited a clear synergistic relationship between spin state switching and luminescence. The complex showed a gradual, incomplete SCO transition between 100 K and 400 K, with a T_1/2_ of 293 K. Simultaneously, the luminescence intensity increased significantly during the LS–HS transition (Figure 22), deviating from the usual thermal quenching of fluorescence. This direct coupling was attributed to an optimized spatial arrangement that enhanced energy transfer between the Fe(II) ion and the AIE-active ligand, unlike earlier complexes [65] where such interactions were hindered by large Fe–tpe separations. Additionally, the absence of π···π interactions between adjacent tpe units in the crystal packing reduced fluorescence quenching, leading to strong solid-state emission. This study represents a step forward in the development of multifunctional SCO-fluorescent materials, demonstrating that careful molecular design can enable strong spin state-dependent luminescence, a crucial property for future switchable photomagnetic materials.

In 2023, the same group extended this approach to naphthalene-decorated **bpp-Nap** (Figure 21) and pyrene–vinyl-decorated **bpp-Py7** (Figure 15) ligands [67]. Two complexes, [Fe(**bpp-Nap**)_2_](ClO_4_)_2_ and [Fe(**bpp-Py7**)_2_](ClO_4_)_2_·2.63CH_2_Cl_2_, underwent complete SCO, with T_1/2_ ≈ 366 K for the naphthalene derivative (no hysteresis) and T_1_/*_2_* ≈ 215–222 K (7 K hysteresis) for the pyrene–vinyl complex. Both were fluorescent, but only in the naphthalene-based system the luminescence was notably enhanced upon entering the HS state, indicating a measurable synergy between SCO and emission. In contrast, the pyrene–vinyl complex showed independent fluorescence and SCO, with the emission varying monotonically with temperature rather than mirroring the spin transition. This comparison highlighted how subtle ligand modifications, even within the same general “**bpp** + polyaromatic” design, can yield stark differences in luminescent–SCO coupling.

Across all these functionalizations—pyrene, fullerene, helicene, phosphazene, radical, and AIE-type substituents—the **bpp** core remains a versatile platform for regulating coordination geometry, spin state properties, and photophysics. For many Fe(II) derivatives, SCO is retained despite the addition of large π-systems, bulky chiral frameworks, or photoactive groups, although achieving a strong synergy between SCO and luminescence or electrical conductivity often demands careful spatial arrangement and electronic matching. In other systems such as Ru(II), Re(I), or Ln(III) complexes, the **bpp** scaffold helps tailor redox or luminescent properties without the possibility of SCO, underscoring its broader applicability to photonic and chiroptical materials. Recent advances in single-molecule electronics, chiral luminescence, and AIE confirm that **bpp**-based designs can adapt to a remarkable spectrum of functionalities. Further structural refinements, especially those decreasing the metal–chromophore distance or enhancing cooperative packing, will likely improve the prospects for robust spin–luminescence or spin–conductance bifunctionality in future materials.

## 5. TTF-Functionalized bpp Complexes

**Figure 23:**
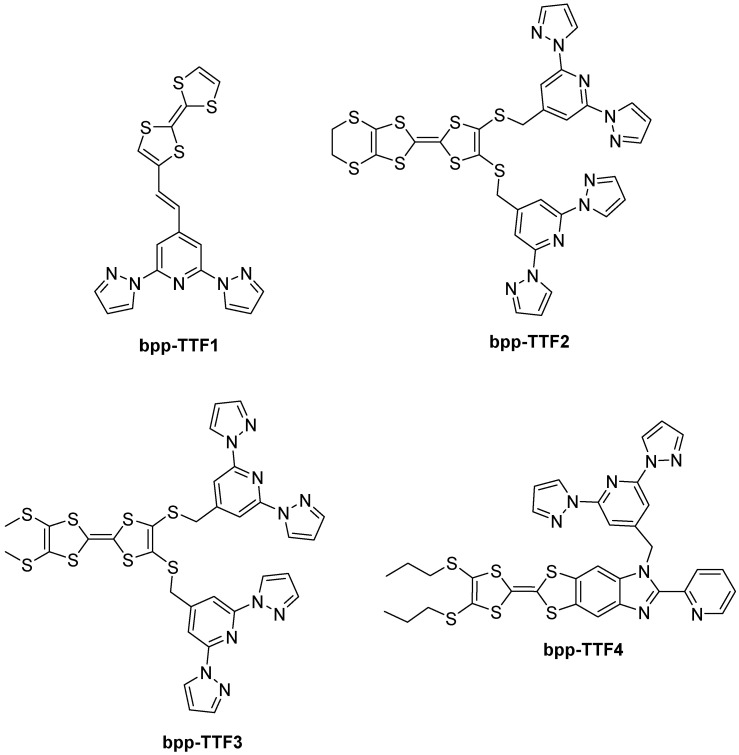
Structures and abbreviations of the TTF-containing ligands discussed in Section 5.

The exploration of **bpp**-type ligands bearing TTF functionalities has led to a broad spectrum of coordination compounds exhibiting diverse physical properties that range from SCO to SMM, luminescence, and even electrical conductivity. The design strategy generally involves merging the **bpp** core, known for supporting SCO in suitable metal complexes, with TTF-based fragments capable of redox activity and intra-ligand charge transfer (ILCT) processes. This chapter provides a detailed chronological review of key examples where **bpp** ligand derivatives, functionalized with TTF units (Figure 23), have been coordinated to different metal centers—predominantly iron(II) for SCO–conductivity synergy and lanthanide ions for SMM and luminescent properties.

### 5.1. **bpp-TTF1**: Merging TTF with **bpp** in Fe(II) Complexes

The first notable demonstration of merging a **bpp**-type ligand with TTF units came in 2011 with the research by Nihei et al. [68]. In that study, the authors synthesized two Fe(II) complexes based on a **bpp** ligand connected to a TTF fragment through ethylene bridge, termed **bpp-TTF1**. One of these complexes, [Fe(**bpp-TTF1**)_2_](BPh_4_)_2_·MeNO_2_·0.5Et_2_O, showed a gradual SCO around 200 K, transitioning from HS Fe(II) at higher temperatures to LS Fe(II) at lower temperatures, whereas [Fe(**bpp-TTF1**)_2_][Ni(mnt)_2_]_2_(BF_4_)·PhCN (mnt = maleonitriledithiolate) had a broader spin transition in the approximately 170–300 K range. Importantly, the [Fe(**bpp-TTF1**)_2_][Ni(mnt)_2_]_2_(BF_4_)·PhCN complex displayed a semiconducting behavior with a room-temperature conductivity on the order of 2.6 × 10^−3^ S cm^−1^. The authors proposed that the SCO-induced structural reorganization modified the conduction pathways in the crystal lattice, thus revealing a synergy between the spin state switching and electrical conduction. This was a significant novelty at the time because it showed how the same material can host redox-active TTF units for semiconducting functionality and an Fe(II) center undergoing a spin transition, effectively coupling the two properties in a single compound.

### 5.2. **bpp-TTF2** and **bpp-TTF3**: Lanthanide Coordination and Luminescence

Subsequent work expanded the TTF–**bpp** concept to lanthanide coordination, aiming to exploit both the redox and ILCT properties of TTF in combination with the high magnetic anisotropy and optically active states of lanthanide ions. In 2014, Feng et al. demonstrated for the first time that TTF-based chromophores can sensitize both visible and NIR lanthanide luminescence [69]. In their study, they developed a novel ligand—**bpp-TTF2**—which is based on TTF and functionalized with bis(2,6-di(pyrazol-1-yl)-4-methylthiolpyridine)-4′,5′-ethylenedithiotetrathiafulvene moieties that provide two distinct coordination sites. Complexes of the general formulas [Ln_2_(hfac)_6_(**bpp-TTF2**]·C_6_H_14_ and [Ln_2_(tta)_6_(**bpp-TTF2**)]·2CH_2_Cl_2_ (Ln(III) = Eu, Tb, Dy, Er, Yb; hfac^−^ = 1,1,1,5,5,5-hexafluoroacetylacetonate anion and tta^−^ = 2-thenoyltrifluoroacetonate) were then investigated, revealing intense Eu(III) luminescence with a lifetime of 0.49 ms. In lanthanide-based systems, as in Fe(II)–**bpp** complexes, the **bpp** ligand maintains its tridentate pincer coordination mode. However, the larger ionic radii and higher coordination number preferences of lanthanides result in a distinctly different overall coordination environment. While Fe(II) typically adopts an octahedral geometry, lanthanides require additional ligands, such as hfac^–^ and tta^–^ in this case, to satisfy their higher coordination demands. This structural adaptability significantly influences the luminescent and electronic properties of the resulting complexes. Upon light irradiation, the coordination complexes demonstrate that ligand **bpp-TTF2** exhibits multiple emission centers, with fluorescence primarily originating from the donor (TTF core) and acceptor (**bpp** unit) groups at room temperature. Additionally, **bpp-TTF2** serves as an organic chromophore that sensitizes the luminescence of lanthanide ions. The novelty lay in the fact that TTF ligands had most often been used to sensitize NIR emissions [70], whereas this work demonstrated visible Eu(III) emission sensitization. This novel sensitization is achieved via an efficient energy transfer from the ligand’s triplet state to the Eu(III) ion, enabling the visible luminescence emission typically observed in high-symmetry Eu(III) complexes. The studied complexes effectively demonstrate the potential of integrating TTF cores with **bpp**-derived coordination sites to achieve new optical functionalities.

Two years later, in 2016, the same group of Feng et al. reported dysprosium- and ytterbium-based complexes [71] involving new TTF-derivative **bpp-TTF3** and an already described ligand **bpp-TTF2** [69]. Their compounds included [Dy_2_(hfac)_6_(**bpp-TTF3**)]**·**C_6_H_14_, [Dy(hfac)_3_(**bpp-TTF2**)]·CH_2_Cl_2_, and [Yb(hfac)_3_(**bpp-TTF2**)]·CH_2_Cl_2_. In these systems, complex [Dy(hfac)_3_(**bpp-TTF2**)]·CH_2_Cl_2_ with the most regular coordination geometry (C_4v_) emerged as a field-induced SMM, revealing frequency-dependent out-of-phase signals in the alternating current (ac) susceptibility under an applied direct current (dc) field. This finding tied in well with prior observations of TTF-based Dy(III) SMMs. By contrast, complexes [Dy_2_(hfac)_6_(**bpp-TTF3**)]**·**C_6_H_14_ and [Yb(hfac)_3_(**bpp-TTF2**)]·CH_2_Cl_2_ did not show slow relaxation in zero field under the same conditions, underscoring how small structural and electronic variations, such as differences in coordination geometry, can drastically change the relaxation dynamics of Dy(III) or Yb(III).

### 5.3. **bpp-TTF4**: Multifunctional Ligands for Lanthanide SMMs

In 2015, the group of Cador and Ouahab explored the SMM properties of dysprosium dinuclear complexes incorporating a new multi-functionalized TTF-based ligand, **bpp-TTF4** [72]. This ligand is engineered with two distinct coordination sites—a bidentate benzimidazol-2-ylpyridine (bzip) unit and a tridentate **bpp** moiety. Two different complexes, [Dy_2_(hfac)_6_(**bpp-TTF4**)]·(CH_2_Cl_2_)_2_·C_6_H_14_ and [Dy_2_(hfac)_3_(tta)_3_(**bpp-TTF4**)], were synthesized. The Dy(III) centers in these compounds occupied different coordination geometries—one center adopting D_4d_ symmetry and the other D_3h_—leading to distinct relaxation dynamics. Ac magnetic susceptibility measurements revealed slow relaxation in zero dc field, and when an external field was applied, the system showed multiple relaxation processes attributable to the distinct dysprosium environments. These findings demonstrated how TTF-containing ligands can stabilize Dy(III) centers in geometries conducive to SMM behavior, pointing toward a versatile strategy to build polynuclear lanthanide complexes for magnetic applications.

Building on the previously reported synthesis of the multichelating ligand **bpp-TTF4** [72], in 2017, the same group introduced a new dinuclear Yb(III) complex, [Yb_2_(hfac)_6_(**bpp-TTF4**)]·2(CH_2_Cl_2_)·C_6_H_14_ [73]. In this complex, **bpp-TTF4** coordinates two Yb centers through its two distinct chelating sites, resulting in two different coordination geometries—an eight-coordinate square antiprism and a nine-coordinate spherical tricapped trigonal prism. The complex efficiently sensitizes Yb-centered luminescence via its lowest-energy ILCT band, with the two Yb(III) ions exhibiting distinct emission profiles that reflect their unique coordination environments. This work not only underscores the versatility of **bpp**-derived frameworks in lanthanide coordination chemistry, but also demonstrates how TTF-based systems can be tailored to achieve innovative optical functionalities.

### 5.4. Heterometallic 3d–4f and 4f–4f′ Complexes via **bpp-TTF4**: Advanced Multifunctionality

**Figure 24:**
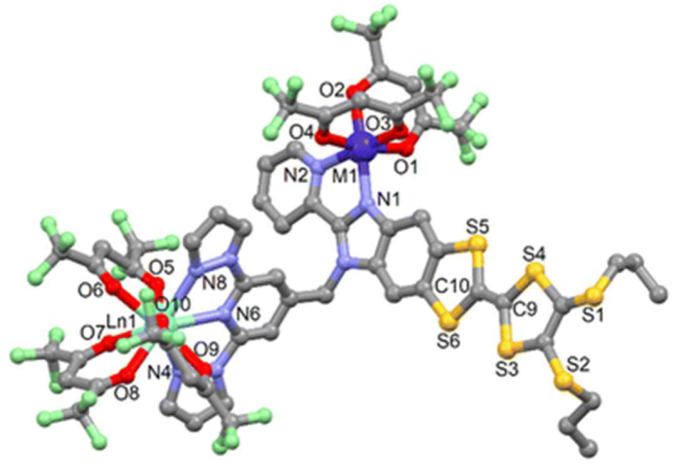
Representative molecular structure of the series of compounds with the **bpp-TTF4** ligand (M(II) = Cd, Zn, Mn, Co, and Ni; Ln(III) = Dy and Yb). Color code: M(II) = violet, Ln(III) = purple, C = gray, N = blue, O = red, S = yellow. Hydrogen atoms and solvent molecules of crystallization are omitted for clarity. Reprinted from [74] under a Creative Commons Attribution License (CC-BY).

The most recent advancement covered here comes from a 2022 study by the aforementioned group, which reports the design, synthesis, and comprehensive characterization of heteroleptic dinuclear complexes featuring both 3d and 4f (as well as 4f–4f′) metal centers using multifunctional ligand **bpp-TTF4** [74]. The inherent difference in denticity and coordination preferences allows for selective binding of a transition metal (M(II) = Cd, Zn, Co, Mn, Ni) to the bzip site and a lanthanide ion (Ln(III) = Dy, Yb, Nd) to the **bpp** site. The resulting 3d–4f complexes, formulated as [MLn(hfac)_5_(**bpp-TTF4**)]_n_ (with additional solvate molecules), are fully characterized by single-crystal X-ray diffraction (Figure 24). Structural analysis confirms that the M(II) ion adopts an N_2_O_4_ coordination sphere, the Ln(III) ion—an N_3_O_6_ environment. To further extend the methodology, the authors develop a strategy for assembling heterobimetallic 4f–4f’ complexes. Here, coordination selectivity is based on differences in ionic radii: a larger lanthanide (e.g., Nd(III) or Pr(III)) selectively occupies the **bpp** site, while a smaller lanthanide (e.g., Dy(III) or Yb(III)) is directed to the bzip site. This stepwise approach, combined with modifications of ancillary ligands (using mixtures of hfac^−^ and tta^−^), enhances the discrimination between coordination sites and yields complexes with well-defined metal distributions, as confirmed by energy dispersive spectrometry and single-crystal studies.

Electrochemical studies by cyclic voltammetry reveal that the redox activity of the TTF core of **bpp-TTF4** is retained upon complexation, with two successive one-electron oxidations corresponding to the formation of a radical cation and dication. The UV–visible absorption spectra (recorded both in solution and in the solid state) display a low-energy ILCT band, red-shifted upon coordination, along with transitions attributable to intra-TTF, intra-hfac^−^, and intra-**bpp** processes. Notably, photophysical studies demonstrate that Yb(III)-containing 3d–4f complexes exhibit NIR luminescence (2F_5/2_ → 2F_7/2_ transition) whose intensity is highly sensitive to the nature of the coordinated transition metal. Complexes featuring a closed-shell Zn(II) show intense emission, while those with paramagnetic ions such as Co(II) or Ni(II) exhibit quenched luminescence; partial quenching is observed with Mn(II). In the 4f–4f′ series, selective energy transfer processes give rise to Nd(III) or Yb(III) NIR emissions depending on the metal pairing, and one compound even demonstrates efficient Nd-to-Yb energy transfer. Static and dynamic magnetic measurements provide evidence for field-induced SMM behavior in the Dy(III)-containing complexes. Detailed magnetic characterization, including temperature-dependent susceptibility and ac magnetometry, reveals that the coordination environment (e.g., eight- versus nine-coordinate Dy(III) centers) and the nature of the associated 3d ion (which affects dipolar interactions and local crystal fields) subtly modulate the magnetic relaxation dynamics. In several compounds, the slow magnetic relaxation is attributed to the combined effects of high magnetic anisotropy at the Dy(III) centers and the tailored coordination sphere provided by **bpp-TTF4**. Moreover, one highlighted complex ([Dy_1.21_Nd_0.79_(hfac)_6_(**bpp-TTF4**)]·2CH_2_Cl_2_·C_6_H_14_) uniquely displays both NIR luminescence and slow magnetic relaxation, qualifying it as a field-induced luminescent SMM. Overall, the study demonstrates that the use of a dual-site ligand with distinct chelating groups enables selective coordination of 3d and 4f ions (or two different 4f ions) by exploiting differences in coordination number and ionic radius. This strategic design yields heterobimetallic complexes that combine enhanced magnetic properties (SMM behavior) with tunable photophysical characteristics (NIR luminescence), underscoring their potential for applications in high-density data storage, quantum computing, and advanced luminescent devices.

Taken together, these studies demonstrate how **bpp**-type ligands decorated with TTF moieties serve as multifunctional modules in coordination chemistry. Early work on Fe(II) complexes established that **bpp-TTF1** can enable both SCO and conductivity in a single molecule, with the synergy manifesting as spin state changes that modify conduction pathways. Later developments expanded toward lanthanide systems, showcasing the coupling of TTF redox and ILCT properties with the unique magnetic anisotropy and optically active states of Dy(III), Eu(III), Yb(III), and other lanthanides. The ability of the TTF core to facilitate efficient ILCT gives rise to luminescence sensitization, while at the same time the **bpp** coordination site locks rare-earth ions into geometries that can exhibit SMM behavior. In certain cases, these dual functionalities operate in tandem, as evidenced by Dy(III) complexes that manifest slow magnetic relaxation and simultaneously display luminescent properties. The novelty repeatedly highlighted in these works includes the first demonstration of SCO–conduction synergy in a Fe(II)–**bpp-TTF1** system, the unprecedented visible Eu(III) luminescence sensitized by a TTF-based chromophore, and the various fine-tuned SMM behaviors controlled by the geometry around Dy(III) ions. Such progress points to the broader promise of using TTF-functionalized **bpp** frameworks to design next-generation coordination materials where spin state transitions, luminescence, conduction, and magnetic blocking may be precisely orchestrated.

## 6. Surface Anchoring and Extended Conjugation in bpp-Based Materials

**Figure 25:**
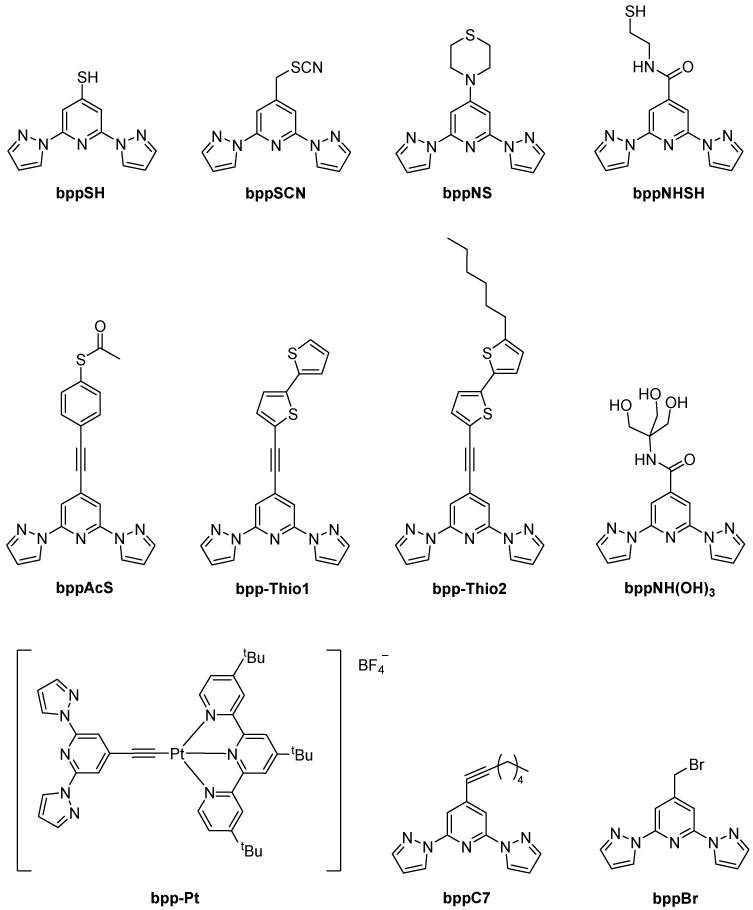
Structures and abbreviations of the ligands discussed in Section 6.

This chapter continues to explore how targeted modifications at the 4-position of the **bpp** ligand (Figure 25) can profoundly influence the structural, magnetic, and electronic properties of the resulting complexes. We begin by examining sulfur-functionalized derivatives that enable robust anchoring on surfaces (e.g., Au(111) and highly oriented pyrolytic graphite, HOPG) and facilitate the formation of well-ordered SAMs. Subsequent sections expand the discussion to derivatives bearing extended thiophene and alkynyl substituents, which introduce conjugation and flexibility into the ligand framework. In addition, halomethyl-functionalized **bpp** derivatives and **bpp** units integrated into polyoxometalate (POM) frameworks are reviewed, underscoring how even subtle changes in ligand substitution can lead to dramatically different magnetic phenomena, luminescence properties, and responses to external stimuli.

### 6.1. Sulfur-Functionalized **bpp** Derivatives for Surface Anchoring

One of the earliest examples of using a sulfur-functionalized **bpp** ligand on surfaces is found in the work of Shen et al. published in 2008 [75]. Here, a **bppNHSH** ligand was synthesized through the formation of an amide linkage to introduce a thiol terminus. The authors demonstrated that highly ordered SAMs could be formed on Au(111) substrates, especially when a perylene-3,4,9,10-tetracarboxylic acid dianhydride (PTCDA) precoating was applied. Scanning tunneling microscopy (STM) revealed well-defined domains with a $\sqrt{3}\times 5$ unit cell, while X-ray photoelectron spectroscopy (XPS) confirmed robust Au–S binding and minimal sulfur oxidation. Near-edge X-ray absorption fine structure spectroscopy (NEXAFS) supported an upright orientation of the **bpp** moiety with estimated tilt angles for the pyridine and pyrazole rings. This work highlighted a potential route to metal-coordination platforms or SCO-active surfaces, although the authors noted that the densely packed trans-trans conformation of the **bpp** headgroup could hinder subsequent complexation on-surface.

A continuation of interest in surface chemistry and **bpp**-type ligands came from the same broader research network, including Grohmann et al. in 2010 [76]. That study focused on a Fe(II) complex bearing a **bpp**-like ligand with thiocyanate substitution, namely [Fe(**bppSCN**)_2_](BF_4_)_2_, which has an abrupt SCO transition near 272 K in the bulk. When deposited on HOPG, the molecules self-assembled into chain-like clusters. STM and current-imaging tunneling spectroscopy (CITS) measurements clearly distinguished between LS and HS states at the single-molecule level, with the LS state showing a notably higher conductance. Moreover, tiny clusters of the complex occasionally switched spin states during successive scans, suggesting that even small aggregates of **bpp**-based Fe(II) molecules can preserve some cooperative SCO character. This represented a groundbreaking demonstration of direct single-complex identification of LS vs. HS states in a surface environment at room temperature, hinting at possible nanoscale data storage or spintronic applications.

Although not strictly a sulfur-functionalized system, the 2015 work by Pukenas et al. [77] also partially addressed sulfur anchoring strategies. They explored the formation and characterization of iron(II) complexes containing **bpp** ligands, revealing notable nanoscale surface structures and SAMs. Their studies began with the parent complex [Fe(**bpp**)_2_](BF_4_)_2_ drop-cast onto HOPG. Striking “chain-of-beads” patterns, each bead composed of roughly 10–50 intact [Fe(**bpp**)_2_](BF_4_)_2_ molecules, emerged consistently in sizes of 2–6 nm in diameter and up to 10 Å in height. CITS demonstrated two major conduction regimes among these beads, tentatively attributed to local electronic or spin state differences (HS vs. LS); however, the beads largely remained in fixed states, with only rare apparent switching events. XPS of drop-cast films on gold confirmed that the **bpp** framework in [Fe(**bpp**)_2_](BF_4_)_2_ remained chemically intact, showing appropriate elemental ratios of C, N, B, F, and Fe. Despite the solution-state SCO properties of these complexes, the authors noted no obvious thermally or bias-induced switching at the nanoscale, likely due to either the small cluster sizes or the tendency of certain [Fe(**bpp**)_2_]^2+^ salts to adopt Jahn–Teller-distorted geometries that suppress SCO. Beyond the parent compound, functionalized **bpp** ligands such as **bppSH** (4-mercapto-2,6-di(pyrazol-1-yl)pyridine) and **bppNS** (4-(*N*-thiomorpholinyl)-2,6-di(pyrazol-1-yl)pyridine) were examined for SAM formation on gold. While **bppSH** decomposed on gold, yielding elemental sulfur and disrupting the **bpp** core, **bppNS** formed relatively stable SAMs with intact **bpp** rings, though attempts to incorporate iron and produce **bpp**-based SCO monolayers were unsuccessful due to the HS reactivity of the iron(II) centers. These findings collectively underscore both the nanoscale structural versatility of [Fe(**bpp**)_2_]^2+^ and the challenges of preserving SCO behavior and ligand integrity at surfaces.

Building upon the findings of Kershaw Cook et al., a more detailed analysis of specific substituents at the 4-position of the **bpp** ligand reveals distinct electronic influences on the SCO behavior of Fe(II) complexes [44]. The thiol substituent used in the **bppSH** ligand, classified as a weak π-donor, induces only a minor decrease in the T_1/2_ compared to the unsubstituted complex [Fe(**bpp**)_2_]^2+^. This aligns with the observed trend that weak π-bonding substituents exert a limited effect on spin state stabilization. Conversely, the thiocyanomethyl group, incorporating an electron-withdrawing thiocyanate moiety via a flexible methylene linker in the **bppSCN** ligand, exhibits a more pronounced stabilization of the LS, leading to an increased T_1/2_. This effect can be attributed to the inductive electron-withdrawing nature of the thiocyanate group, which enhances Fe(II)–**bpp** π interactions, thereby reinforcing ligand field stabilization. The study further demonstrates that the influence of substituents at the 4-position in **bpp** is dictated by a delicate interplay between metal–ligand σ- and π-bonding effects, with electron-withdrawing groups stabilizing the LS state through the selective stabilization of metal t_2g_ orbitals. These findings underscore the potential of targeted ligand modifications to fine-tune SCO properties, offering valuable strategies for designing Fe(II) complexes with optimized switching behavior for molecular and materials applications.

In parallel, a related sulfur-functionalized derivative (*S*)-(4-{[2,6-(dipyrazol-1-yl)pyrid-4-yl]ethynyl}phenyl)ethanethioate (**bppAcS**)—an acetate-protected thiol on the **bpp** scaffold—was investigated by Devid et al. in 2015 [78]. Here, the Fe(II) complex, referred to as [Fe(**bppAcS**)_2_](ClO_4_)_2_ in bulk form, was used to bridge ~8.5 nm diameter gold nanoparticles in 2D arrays. Raman spectroscopy and superconducting quantum interference device (SQUID) magnetometry confirmed a partial SCO near 270–290 K within the arrays, while electrical measurements revealed non-monotonic temperature-dependent conductance, contrasting with monotonic behavior in reference arrays. Modeling suggested that the HS state is more resistive, creating a shallow conductance minimum around the T_1/2_. This study illustrated that SCO properties can indeed persist in extended nanoparticle networks, giving rise to observable changes in electrical transport upon thermal spin switching.

### 6.2. **bpp** Derivatives with Extended Thiophene Substituents

Expanding the concept of “sulfur-functionalization” to include conjugated thiophene–alkynyl units, Kuppusamy et al. reported two supramolecular Fe(II) **bpp** complexes in 2022 [79]. The ligands 4-([2,2′-bithiophen]-5-ylethynyl)-2,6-di(1*H*-pyrazol-1-yl)pyridine (**bpp-Thio1**) and 4-(2-(5-(5-hexylthiophen-2-yl)thiophen-2-yl)ethynyl)-2,6-di(1*H*-pyrazol-1-yl)pyridine (**bpp-Thio2**) contained thiophene-based arms designed to anchor in break-junction experiments. In bulk form, one complex showed a gradual SCO near 254 K, while the other had a more complex, multistep transition around 356 K. Theoretical DFT modeling indicated that physically stretching the Fe–**bpp** core could favor an HS configuration and thus alter the electrical conductance dramatically. Although mechanical break-junction measurements did not conclusively capture a spin state-dependent conductance switch—likely due to suboptimal molecular orientation—the work offered a valuable blueprint for future single-molecule spintronics, underscoring that suitably engineered **bpp**-thiophene complexes can, in principle, undergo mechanically driven SCO and concurrent transport modulation.

### 6.3. **bpp** Derivatives with Alkynyl Substituents

Moving from surface functionalization to discrete molecular magnets, Ruben and R. Boča group reported a pentacoordinate Co(II) complex based on a **bpp** derivative containing a hept-1-ynyl substituent, termed **bppC7**, in 2014 [80]. The complex [Co(**bppC7**)Cl_2_] features a five-coordinate geometry and displays SMM behavior, as evidenced by ac magnetic susceptibility experiments showing frequency-dependent out-of-phase signals. Remarkably, despite a usually negative zero-field splitting being favored in many SMMs, this system’s positive D-value (~150 cm^−1^) did not preclude slow magnetic relaxation. The authors detected two distinct relaxation processes, likely arising from local anisotropy and dimeric packing via short π–π contacts (~3.4 Å). This result broadened the design landscape for 3d SMMs by highlighting that pentacoordinate Co(II) with an alkynyl-bearing **bpp** can exhibit robust magnetization relaxation.

**Figure 26:**
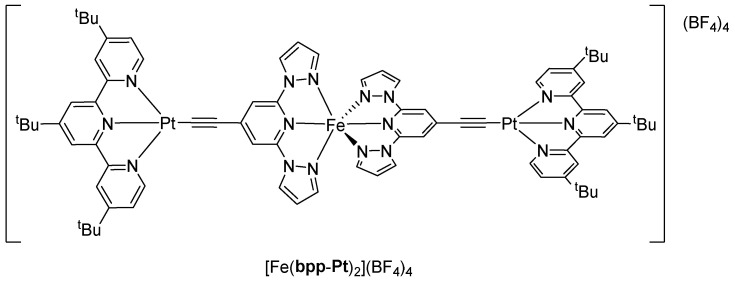
Structure of the [Fe(**bpp–Pt**)_2_](BF_4_)_4_ complex [81].

An expanded approach to alkynyl substitution was undertaken by Schäfer et al. in 2017 [81]. They synthesized a trinuclear complex [Fe(**bpp–Pt**)_2_](BF_4_)_4_ (Figure 26), where “**bpp–Pt**” denotes a Pt(II)-terpy fragment linked through alkynyl moieties to a **bpp**-based core. Two crystalline variants were obtained, differing mainly in solvent content: [Fe(**bpp–Pt**)_2_](BF_4_)_4_·3.5CH_2_Cl_2_ in $P\bar{1}$ and [Fe(**bpp–Pt**)_2_](BF_4_)_4_·10CH_2_Cl_2_ in *C*2/c. Complex [Fe(**bpp–Pt**)_2_](BF_4_)_4_·10CH_2_Cl_2_ exhibited a reversible SCO transition at 268 K, while [Fe(**bpp–Pt**)_2_](BF_4_)_4_·3.5CH_2_Cl_2_ stayed in the HS state across the measured temperature range. Mössbauer spectroscopy confirmed the thermal spin switch in [Fe(**bpp–Pt**)_2_](BF_4_)_4_·10CH_2_Cl_2_ and showed that red light could induce a metastable LS population via LIESST. Photophysically, the Pt(II)-centered phosphorescence was strong in solution but decreased in the solid state, and an efficient quenching channel to the Fe(II) center was noted in ultrafast spectroscopy. Intriguingly, the luminescence did not strongly correlate with the spin state in the solid, reflecting a relatively weak coupling between the Pt(II) chromophores and the SCO-active Fe(II) site. Nonetheless, this remains a rare example of a luminescent multinuclear Fe(II) SCO system, demonstrating how delicate structural packing can toggle spin state behavior.

### 6.4. Halomethyl-Functionalized **bpp** Derivatives

Functionalization at the 4-position of the **bpp** in the pyridine ring with a bromomethyl substituent was explored by the group of Pointillart in 2018 [82]. The same paper discussed both Fe(II) and Co(II) complexes of the ligand 2,6-di(pyrazol-1-yl)-4-(bromomethyl)pyridine (**bppBr**). For Fe(II), the authors synthesized [Fe(**bppBr**)_2_](BF_4_)_2_ in two solvation states: a solvent-free form and a nitromethane-solvated form ([Fe(**bppBr**)_2_](BF_4_)_2_·4MeNO_2_). Both displayed a remarkably HS T_1/2_ (~324–340 K), with abrupt transitions and a substantial hysteresis window (4–6 K for the solvent-free form and up to ~35 K for the solvated form). The solvated phase even showed multistep transitions—up to six distinct steps—indicating a pronounced sensitivity of SCO to crystal packing and solvation. Moreover, a robust LIESST effect allowed photoinduced trapping of the HS state below 55 K. In contrast, the analogous Co(II) complex, [Co(**bppBr**)_2_](BF_4_)_2_·2MeNO_2_, remained in an HS state at all temperatures but exhibited field-induced SMM behavior. Ac susceptibility under a 1000 Oe dc field revealed slow magnetic relaxation with a barrier of 14.6 cm^−1^. This dual outcome—one metal leading to high-temperature SCO and another to SMM—demonstrated how a single **bpp** derivative can support markedly different magnetic phenomena depending on the metal center.

### 6.5. **bpp** Integrated into POM Frameworks

An entirely different but equally intriguing functionalization strategy was presented by the group of Coronado in 2023 [83]. They constructed a one-dimensional hybrid coordination polymer in which hexavanadate POM clusters are alternately bridged by Fe(II)–**bppNH(OH)_3_** units. The resulting Fe–POM chains showed abrupt SCO transitions with hysteresis, the T_1/2_ shifting from ~140 K to ~260 K depending on the solvent in the channels (MeCN vs. MeNO_2_ vs. EtOH). Notably, the hexavanadate POM is itself redox-active, allowing an additional level of tuning via external electron donors or acceptors. The authors also documented a robust LIESST effect, enabling photo-switching of the spin state at low temperatures. This system stands out as the first time a POM-based architecture displayed a complete and abrupt SCO, thus marrying the redox tunability of POMs with the bistable spin states of Fe(II)–**bpp** complexes in one multifunctional material.

These studies collectively illustrate the tremendous flexibility of **bpp** derivatives in designing materials and complexes with varied functionalities—from surface-bound SCO layers and nanoparticle arrays to SMM, luminescent multinuclear complexes, and multifunctional coordination polymers. Introducing substituents such as thiols, alkynyl groups, halomethyl units, or extended conjugated fragments can profoundly influence the ligand field around the metal center, the assembly behavior on surfaces, or the coupling to external stimuli (light, redox agents, mechanical stress). In particular, the synergy of SCO with conductivity, magnetism, or luminescence offers fertile ground for molecular devices at the nanoscale. The reported examples underscore both the promise and the challenges: while many **bpp**-based systems retain structural integrity when grafted onto surfaces or embedded in arrays, achieving complete, robust, and fully switchable functions (e.g., spin transitions triggered by voltage or light in a single-molecule junction) often demands careful tuning of the coordination environment and lattice or surface interactions. Nevertheless, hysteresis widths, magnetic anisotropies, and optical properties highlight the continued potential of **bpp** ligands as a cornerstone of modern spintronic and multifunctional materials research.

## 7. “Back-to-Back” bpp Derivatives and their Functional Architectures

**Figure 27:**
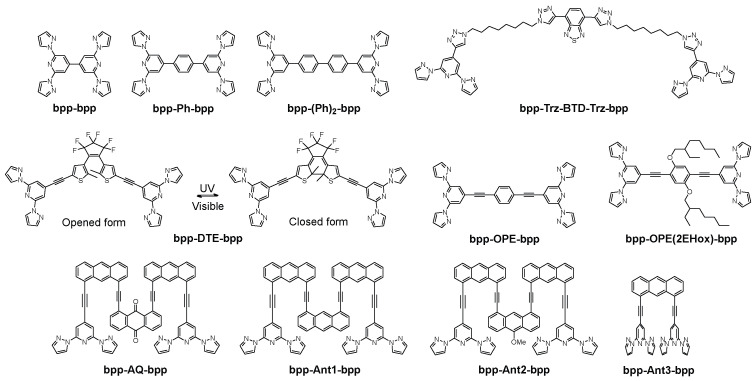
Structures and abbreviations of the “back-to-back” **bpp** derivatives discussed in Section 7.

Within the realm of **bpp** chemistry, “back-to-back” or “extended” **bpp**-based ligands have played a pivotal role in advancing supramolecular and nanoscale device applications. By tailoring the bridging units attached to the **bpp** core—ranging from direct **bpp**–**bpp** connections to phenylene- and anthracene-based spacers (Figure 27)—researchers have engineered a rich diversity of structures with properties as varied as SCO, luminescence, photoswitching, and waveguiding.

### 7.1. **bpp–bpp** Derivatives

A simple yet powerful approach to constructing extended **bpp**-based systems involves directly coupling two **bpp** units in a “back-to-back” fashion without additional aromatic spacers. One of the earliest illustrations is the work by Basak and Chandrasekar published in 2011 [84]. They synthesized a “**bpp–bpp**” ligand designed to self-assemble into blue-emitting nanotubes tens of micrometers long. The core innovation was a one-pot procedure where the rectangular organic nanotubes initially formed via hydrogen bonding and π–π stacking among the **bpp–bpp** units, after which a red-emitting Eu(tta)_3_ complex was added in situ, obtaining [(Eu(tta)_3_)_2_(**bpp–bpp**)]. The Eu complex coordinated to surface **bpp** sites, generating a thin red-luminescent coating on top of the blue-fluorescent **bpp–bpp** interior. This hybrid architecture produced a tricolor emission (blue, red, and purple, a combination of two colors) upon single-wavelength excitation. A key finding was that simply varying the ratio of the Eu complex enabled controllable color changes, illustrating how metal coordination on an organic scaffold can tune emission profiles. The authors noted that these parallelepipedic nanotubes were robust, surviving manipulation in solution and retaining their self-assembled form. The novelty lay in achieving multicolor luminescence from a single, stepwise self-assembly, making the system an embryonic “nanoscale optical device”.

Building on these tubular architectures, the same group in 2014 [85] demonstrated how the same **bpp–bpp** nanotubes functioned as passive optical waveguides. By directing laser light into one end of the tube, they showed minimal attenuation of the transmitted beam along tens of micrometers. Furthermore, the authors used laser ablation to “cut” the tubes with high spatial precision, effectively creating multiple outlets for the guided light. This technique allowed real-time control over the waveguide length and introduced the possibility of multichannel splitting from a single tubular structure. They emphasized the novelty of systematically adjusting the waveguiding path in an organic nanostructure, as well as creating multiple emission ports on a single fiber. The tubes remained operational even off-substrate (suspended in air), corroborating true waveguiding independent of external support. Together, these two papers on the **bpp–bpp** system outlined an elegant route to multicolor luminescence and waveguiding functionalities, firmly establishing the importance of a straightforward “back-to-back” **bpp** derivative in photonic nanodevice research.

### 7.2. **bpp–Ph–bpp** and **bpp–(Ph)_2_–bpp** Derivatives

An alternative approach to extending the **bpp** motif is to incorporate a phenylene ring between two **bpp** units, yielding 1,4-bis(1,2′:6′,1″-bispyrazolylpyridin-4′-yl)benzene (**bpp–Ph–bpp**). This structural motif often facilitates coordination polymers or multinuclear architectures, owing to the longer spacer and additional π-conjugation. One of the earliest examples is the 2007 study by the group of Ruben [86]. They presented a linear Fe(II) chain, [Fe(**bpp–Ph–bpp**)]_n_(BF_4_)_2n_, exhibiting an SCO above room temperature. The spin transition occurred around 328 K (on heating) and 318 K (on cooling), with a notable 10 K hysteresis, reflecting cooperative effects along the 1D chain. Although the material was insufficiently soluble for single-crystal X-ray studies, mass spectrometry confirmed a linear polymeric arrangement. The authors underscored the significance of shifting the SCO temperature above ambient conditions, highlighting potential applications in molecular switches or sensors that operate near everyday temperatures.

Two years later, the same group expanded the scope of the **bpp–Ph–bpp** chemistry to ruthenium complexes [87]. They reported both mononuclear and binuclear Ru(II) complexes bearing terpy and **bpp–Ph–bpp** units, namely [(terpy)Ru(**bpp–Ph–bpp**)](PF_6_)_2_ and [(terpy)Ru(**bpp–Ph–bpp**)Ru(terpy)](PF_6_)_4_. Structural data revealed how the smaller bite angle of the **bpp** moiety imposed slightly constrained Ru coordination geometries. Both complexes displayed MLCT absorption bands (e.g., 443 nm for the terpy-based transition in the mononuclear species) and luminescence at low temperatures (around 600–620 nm). Notably, at room temperature, the emission was largely quenched, but at 77 K, photoluminescence became readily observable. The binuclear complex also exhibited redox events, suggesting weak electronic communication between the two Ru centers through the **bpp–Ph–bpp** bridge. The novelty of this work lay in demonstrating that **bpp–Ph–bpp** can bridge multiple metal centers while retaining luminescence and distinct electrochemical profiles, thereby paving the way for more elaborate polynuclear arrays.

In 2010, attention to **bpp–Ph–bpp** continued in the self-assembly study by Chandrasekhar and Chandrasekar [88]. Although this work primarily contrasted two ligands—one bridging the **bpp** units via a single phenylene (**bpp–Ph–bpp**), another—via biphenyl (**bpp–(Ph)_2_–bpp**)—it provided an important demonstration of how subtle changes in the bridging spacer can alter self-assembled shapes. The **bpp–Ph–bpp** system formed elongated nanotapes emitting in the violet region, whereas the biphenyl-bridged derivative folded into submicrometer-scale tubes that strongly fluoresced in the violet range. These morphological differences were attributed to variations in hydrogen bonding and π–π stacking as well as the differing solubility profiles. The authors noted that **bpp–Ph–bpp** nanotapes and **bpp–(Ph)_2_–bpp** submicrotubes could find use in miniaturized luminescent scaffolds, leveraging the strong violet fluorescence for sensing or device integration.

In 2013, the same group further explored submicrotubes of **bpp–(Ph)_2_–bpp** [89]. They acted as efficient optical waveguides capable of transporting laser light over tens of micrometers. The authors demonstrated multiple geometric configurations (linear, bent, crossed, tip-to-tip) and reported that waveguiding worked even for tubes suspended in air, confirming total internal reflection within the organic walls. A key highlight was “remote excitation”, where a fluorophore placed tens of micrometers away could be excited by light traveling along the **bpp–(Ph)_2_–bpp** tube. This pointed to the potential for integrated photonic circuits wherein active and passive elements (light sources, sensors, couplers) can be assembled from purely organic crystals.

A series of subsequent studies by the aforementioned group showcased novel functionalities derived from **bpp–(Ph)_2_–bpp** tubes. In 2016, they reported a transformation of an active-type photonic resonator into a passive-type heterostructure at the single-particle level [90]. This study explored the self-assembly of **bpp**-based π-conjugated molecules into crystalline hexagonal submicrotubes with whispering gallery mode (WGM) resonances in the visible range (400–600 nm). Upon UV laser excitation, these tubes exhibited a fivefold fluorescence enhancement compared to the powder state due to the photonic cavity effect. A key finding was the laser-induced conversion of active-type homo-structure resonators into passive-type hetero-structures. Intense laser irradiation (42 mW) at the tube ends generated carbonaceous regions, enabling passive WGM resonances in the visible–NIR range (500–750 nm) under visible laser excitation. This dual-functionality—enhanced fluorescence in the active mode and light modulation in the passive mode—offered reconfigurable photonic behavior. The ability to reversibly tune optical properties at the single-particle level highlights the potential of **bpp** derivatives in developing advanced, adaptable photonic devices for sensing, light guiding, and integrated photonic circuits.

Over the next few years, Chandrasekhar’s group exploited the mechanical flexibility of **bpp–(Ph)_2_–bpp** crystals to create complex optical waveguides and multicolor outputs. They described a composite flexible organic crystal waveguide for broadband multiplexed visible-light transport [91]. The **bpp–(Ph)*_2_*–bpp** crystal functioned as a robust “backbone waveguide”, onto which organic microcrystals of (Z)-2-(3,5-bis(trifluoromethyl)phenyl)-3-(7-methoxybenzo[c][1,2,5]thiadiazol-4-yl)acrylonitrile (BTD2CF_3_) and 9,10-bis(bromo)anthracene (DBA) were physically appended, emitting yellow and cyan light, respectively. By leveraging the partial spectral overlap, where the blue emission of **bpp–(Ph)_2_–bpp** excited the adjacent crystals, which subsequently emitted yellow or cyan, a cascaded emission system spanning 420–750 nm was achieved. This multiplexing allowed multiple color channels to propagate simultaneously. The authors emphasized how the mechanical bending of the waveguide did not significantly degrade the optical output, illustrating a real advantage over brittle inorganic fibers.

In 2023, the same group pushed this concept further by developing “amphibian-like” flexible organic crystal fibers that worked both in air and underwater [92]. The **bpp–(Ph)_2_–bpp** rods, again, millimeters long, with a rectangular cross-section, guided visible light with low loss coefficients (e.g., 0.07226 dB μm^−1^ in the straight configuration). Critically, their hydrophobic surfaces and robust mechanical properties meant that waveguiding persisted even when the rods were immersed in water. This feature enabled “amphibian-like” operation, giving the potential for underwater micro-precision sensing or off-shore optical communications. Remote excitation experiments confirmed that fluorescent targets several micrometers away from the waveguide terminus could be lit efficiently, highlighting the synergy between mechanical flexibility, environmental compatibility, and waveguiding efficiency.

Most recently, in 2024, the group of Chandrasekar produced a report called “A Hybrid Organic-Crystal-Based Curved Waveguide Network for Producing, Splitting, and Transducing Multi-Color Outputs” [93]. They integrated two distinct crystals—one based on **bpp–(Ph)_2_–bpp** (blue emission) and another containing a green-emitting naphthalimine-like moiety—into a curved waveguide network. By carefully bending and coupling the two crystals at specific contact points, they produced a 2 × 2 directional coupler supporting multicolor emission splitting. Depending on the input port, the waveguide network could output two or three different color signals simultaneously. Energy transfer from the blue-emissive region (**bpp–(Ph)_2_–bpp**) to the green moiety was leveraged to broaden the overall spectral coverage. This demonstration of multiport, multicolor splitting in a purely organic system represents a significant step toward integrated photonic logic, neural-like networks, and advanced color routing on the microscale.

### 7.3. Amphiphilic **bpp** Derivatives and Triple Emission

In 2011, the same group introduced a different angle on **bpp**-based luminescence by designing an amphiphilic ligand (**bpp–Trz–BTD–Trz–bpp**) carrying multiple emissive fragments [94]. This ligand was synthesized through click chemistry, linking two blue-emitting 4-triazolyl-2,6-bispyrazolylpyridine derivatives to a green-emitting 4,7-triazolyl-benzo-1,2,5-thiadiazole core using two octyl chain linkers. Upon self-assembly in THF/water, this amphiphile formed vesicles that already showed dual-color emission (blue and green) from the organic segments. By coordinating Eu(tta)_3_ moieties to the **bpp** sites on the vesicle membranes, they achieved a red-emitting inorganic layer, thereby yielding a net triple emission (blue, green, and red) under one-wavelength excitation. Remarkably, the transition from dual to triple color was carried out in situ by adding Eu(III) complexes to the same self-assembly, echoing the concept of “one-pot” hybrid formation seen in earlier **bpp–bpp** nanotubes [84], but now in a vesicular form. The authors described this as “unusual and first of its kind” since multicolor organic vesicles, especially ones that incorporate rare-earth complexes for red emission, are rarely realized in a single-step bottom-up protocol. Potential applications range from color barcoding to multichannel fluorescence imaging or white-light generation from a single nanoscale entity.

### 7.4. **bpp**-Based Dithienylethene Photoswitches

Beyond waveguiding and luminescence, **bpp** can also be combined with photoswitchable units such as dithienylethene. In 2015, Chandrasekhar’s group produced a report called “Photonic Microrods Composed of Photoswitchable Molecules: Erasable Heterostructure Waveguides for Tunable Optical Modulation” [95]. There, a dithienylethene core was linked to two **bpp** moieties (a “back-to-back” arrangement) to yield microrod **bpp–DTE–bpp** crystals that waveguided visible light. Under UV irradiation, the dithienylethene switched to its closed form, significantly absorbing the waveguided beam and effectively introducing a time-dependent delay in transmission. The authors exploited this phenomenon to “write” and “erase” optical modulators within the same microrod by local laser activation, creating small closed-state regions that temporarily blocked or delayed light propagation. Upon exposure to visible light, the closed dithienylethene segments reopened, restoring the original waveguiding capacity. This dynamic reconfigurability underscored how **bpp**-based molecular crystals can be engineered not only for static waveguides, but also for real-time photonic modulation, with potential in optical computing or data encoding.

### 7.5. Anthracene-Bridged **bpp** Derivatives

Anthracene-based bridging of **bpp** units enables photoisomerizable scaffolds and polynuclear SCO complexes. In 2017, Šalitroš et al. synthesized a series of ligands featuring two **bpp** units linked via a multi-1,8-diethynylanthracene scaffold, culminating in three isostructural analogues—**bpp–AQ–bpp** (with an anthraquinone unit), **bpp–Ant1–bpp** (with an anthracene unit), and **bpp–Ant2–bpp** (with a 10-methoxyanthracene unit)—obtained in an overall yield of 8–9 % over eight synthetic steps [96]. Notably, under blue-light irradiation (~470 nm) at a low temperature (5 °C), **bpp–Ant1–bpp** and **bpp–Ant2–bpp** underwent a selective [4+4] photocycloaddition, forming bridged anthracene dimers within the same molecule. X-ray crystallography unambiguously confirmed the newly formed C–C bonds (1.61–1.65 Å). Subsequent thermal treatment above ~165–180 °C reversed the cycloaddition, regenerating the original anthracene moieties. This reversible photoreaction was accompanied by pronounced changes in the emission spectra and redox behavior, suggesting potential applications in multistate photo-switchable ligands. In contrast, photoisomerization in **bpp–AQ–bpp** is entirely suppressed, likely due to the inhibition of cycloaddition resulting from its altered light absorption properties. Because each half of the molecule contained a **bpp** site, these ligands could, in principle, support photoresponsive SCO if coordinated to transition metals, bridging the gap between photochemistry and spin state switching.

Building on this concept, the same group further explored the potential of anthracene-bridged **bpp** ligands in polynuclear Fe(II) complexes**.** In 2019, they synthesized polynuclear Fe(II) architectures using **bpp–Ant3–bpp** and **bpp–AQ–bpp** ligands, forming a tetranuclear Fe_4_ grid and a hexanuclear Fe_6_ ring, respectively [97]. One of the resulting complexes, [Fe_4_(**bpp–Ant3–bpp**)_4_](CF_3_SO_3_)_8_·7CH_3_CN, exhibited a partial SCO transition, where two out of four Fe sites switched spin states. In contrast, [Fe_6_(**bpp–AQ–bpp**)_6_](CF_3_SO_3_)_12_·18CH_3_NO_2_·9H_2_O remained in the HS state across the entire temperature range. Despite the face-to-face arrangement of anthracene units within the lattice, no [4+4] cycloaddition occurred. The authors attributed this inactivity to steric constraints and the rigidity imposed by the polynuclear Fe(II) framework in [Fe_4_(**bpp–Ant3–bpp**)_4_](CF_3_SO_3_)_8_·7CH_3_CN as well as the inhibition of photocyclization in [Fe_6_(**bpp–AQ–bpp**)_6_](CF_3_SO_3_)_12_·18CH_3_NO_2_·9H_2_O, consistent with previous studies on the free **bpp–AQ–bpp** ligand [96]. Nonetheless, the incorporation of anthracene introduced interesting photoluminescent traits, and the structural distortions around the metal centers resulted in large zero-field splitting parameters, providing a complex interplay of spin states and potential photoreactivity.

### 7.6. **bpp**–Phenylene Ethynylene Derivatives

Finally, bridging **bpp** units with oligo(p-phenylene ethynylene) (OPE) segments offers yet another avenue to integrate luminescent π-conjugation with SCO-active metal sites. In 2020, the group of Ruben reported two novel ligands, each bearing **bpp** termini and OPE backbones, which were complexed with Fe(II) to yield one-dimensional coordination polymers, albeit insoluble in common solvents [98]. The unsubstituted variant [Fe(**bpp–OPE–bpp**)]_n_(BF_4_)_2n_ displayed a nearly complete spin transition at ~275 K, while the derivative with bulky 2-ethylhexyloxy side-chains [Fe(**bpp–OPE(2EHox)–bpp**)]_n_(BF_4_)_2n_ showed only partial (about 50%) SCO. Both materials also exhibited LIESST at low temperatures, indicating that iron centers in these complexes could be toggled between HS and LS states by light. Interestingly, the free ligands themselves were notably fluorescent (due to the OPE core), but coordination to Fe(II) quenched most of this emission, precluding direct coupling between SCO and photoluminescence. Nonetheless, the design strategy foreshadowed the possibility of future “molecular wire” architectures where spin state switching might be harnessed to modulate conductivity or optical signals along extended π-conjugated backbones.

Taken together, these studies showcase how “back-to-back” **bpp** derivatives, in which two **bpp** moieties are linked through various organic spacers, provide a versatile foundation for next-generation functional materials. By choosing bridging groups that impose distinct π–π interactions, hydrogen-bonding motifs, or steric demands, researchers have accessed a gamut of properties—tricolor luminescence (blue–red–purple), passive and active optical waveguiding with single- or multiport outputs, photoisomerization, high-temperature SCO, and even dynamic photoresponsive modulation.

**Bpp–bpp** architectures exemplify the simplicity and efficiency of constructing multicolor luminescent nanotubes and waveguides. Incorporating phenyl bridges into **bpp–Ph–bpp** derivatives has facilitated SCO near room temperature, led to Ru-based luminescent complexes, and enabled the formation of well-defined self-assembled tapes. Expanding to **bpp–(Ph)_2_–bpp** systems has resulted in a diverse array of photonic devices, including molecular tubes with waveguiding capabilities, resonator transformations, flexible multiplexers, and underwater “amphibian-like” optical fibers. Amphiphilic **bpp** derivatives have demonstrated the power of solvent-driven self-assembly, forming vesicles capable of triple emission through lanthanide coordination. **Bpp**-based dithienylethenes have introduced erasable and reconfigurable optical modulators, expanding possibilities for dynamic photonic applications. Anthracene-bridged **bpp** structures have revealed complex photoinduced cycloadditions and partial SCO in polynuclear grids, while **bpp**–OPE hybrids have opened the door to “molecular wire-like” materials that integrate π-conjugation with SCO functionality.

In sum, the progression from 2007 to the present day underscores an increasing mastery over both synthetic routes and functional integration. Early work established fundamental coordination motifs and spin transitions, followed by a surge of activity in luminescent self-assemblies, waveguides, and photoresponsive designs, culminating in intricate multi-crystal couplings and broad-wavelength multiplexing. This trajectory promises continued innovation in fields such as optical logic, miniaturized sensors, flexible photonic circuits, and advanced SCO devices. By systematically tuning each segment of the “back-to-back” motif—pyrazolyl, pyridyl, and bridging arylene—the **bpp** platform will undoubtedly keep generating new molecular architectures that bridge traditional chemistry and emerging photonic, magnetic, and sensing applications.

## 8. Conclusions and Outlook

Decades of research on **bpp** ligands and their derivatives demonstrate a remarkable breadth of functions—SCO, SMM, luminescence, catalysis, electrochemical energy storage, and photonics—all emanating from strategic substitutions at the pyridine 4-position. The many findings covered in this review firmly establish **bpp** as a central scaffold for constructing advanced coordination materials with tailored physical properties. What sets **bpp** apart is its synergy of synthetic accessibility, robust chelation, and ready functionalization: comparatively minor modifications (for example, linking a carboxyl group, a TTF redox core, extended aromatics, or bridging two **bpp** units “back to back”) can drastically alter spin state transitions, magnetic anisotropy, photoluminescence, conductivity, or redox behavior.

One major direction is the push toward high-temperature spin switching and conductivity. Numerous studies have pushed the SCO transition of iron(II)–**bpp** complexes well above room temperature, in some instances with considerable thermal hysteresis. This paves the way for practical spin-based devices functioning under ambient conditions, such as spintronic memory elements or switchable electrodes. Concurrently, doping strategies (e.g., in RFBs or hole transport layers for perovskite cells) show that cobalt–**bpp** complexes can blend stable redox activity with partial spin switching, offering new avenues for high-voltage battery designs and improved solar cell architectures. However, practical implementation of these materials faces challenges related to long-term stability, device integration, and reproducibility in large-scale fabrication. Addressing these concerns will require interface engineering strategies, such as protective coatings for metal centers, improved ligand design to minimize structural degradation, and hybrid material integration to balance performance with robustness.

Another key development is the design of truly multifunctional materials. Examples include TTF-functionalized **bpp** scaffolds that couple spin state changes to moderate electrical conductance or lanthanide complexes that combine field-induced slow magnetic relaxation with NIR luminescence. Beyond fundamental novelty, these designs open applications in molecular spintronics (where conduction can be gated by spin switching), luminescent SMMs (for magneto-optical data storage), and reconfigurable photonic structures. However, achieving strong metal–ligand electronic coupling and fine-tuned supramolecular packing remains a challenge. Future efforts should focus on modular synthesis strategies, where well-defined molecular building blocks can be systematically combined to control electronic communication, optimize cooperative effects, and simplify synthetic routes for broader accessibility.

A final emergent trend lies in complex photonic architectures and advanced self-assemblies. “Back-to-back” **bpp** derivatives illustrate how entirely organic constructs—sometimes without any coordinating metal center—self-assemble into waveguides, nanotubes, flexible crystal fibers, or vesicular networks that guide light with minimal losses. Demonstrations of multiport color splitting, underwater (“amphibian-like”) waveguiding, and in situ doping with fluorescent metal complexes highlight the adaptability of **bpp**-based materials in advanced photonic circuits. Achieving stable, reconfigurable devices capable of mechanical bending, remote laser “cutting”, or photo-induced luminescence changes will be especially attractive for future integrated photonics or wearable optical sensors. While these advances are promising, practical integration into optical technologies will require scalable processing methods and better environmental resistance, particularly against humidity and temperature variations.

Another exciting avenue is the development of **bpp**-based catalysts for green chemistry applications. While **bpp** ligands have been explored in various catalytic transformations, such as polymerization, oxidation, hydroboration, and cross-coupling [6,7,8,9,10,11,12,13], their potential for sustainable catalysis, including solvent-free reactions, remains an area for further exploration. The modular nature of **bpp** ligands allows for fine-tuning of electronic properties, which could enable selective and recyclable catalytic systems. Future efforts could focus on adapting **bpp**-based catalysts for more environmentally friendly processes, such as ligand frameworks derived from bio-based or recyclable feedstocks, minimizing the reliance on harsh solvents and energy-intensive purification methods.

In short, the **bpp** platform continues to expand its reach into molecular electronics, magnetism, optics, and energy storage, often merging what were once standalone functionalities (such as SCO, redox, or luminescence) into single, integrated architectures. From room-temperature spin switches and luminescent SIMs to water-stable organic waveguides and multicolor laser devices, the progress outlined here underscores that **bpp** is not just a ligand but a versatile “molecular toolbox” for high-performance functional materials. New synthetic approaches that pair **bpp** with modern organic and organometallic fragments—especially those designed to enhance spin–photon or spin–charge coupling—are poised to deliver next-generation systems suitable for spintronic memory, advanced optical circuits, and beyond.

## Data Availability

No new data were created or analyzed in this study. Data sharing is not applicable to this article.
